# Ecological Interactions Affecting the Efficacy of *Aphidius colemani* in Greenhouse Crops

**DOI:** 10.3390/insects6020538

**Published:** 2015-06-11

**Authors:** Sara G. Prado, Sarah E. Jandricic, Steven D. Frank

**Affiliations:** 1 David Clark Labs, Department of Applied Ecology, North Carolina State University, Raleigh, NC 27695, USA; 2 Ontario Ministry of Agriculture, Food and Rural Affairs, 4890 Victoria Avenue North, Vineland, ON L0R 2E0, Canada; E-Mail: Sarah.Jandricic@ontario.ca; 3 Gardner Hall, Department of Entomology, North Carolina State University, Raleigh, NC 27695, USA; E-Mail: sdfrank@ncsu.edu

**Keywords:** tritrophic interactions, aphid biological control, parasitoid abundance, parasitoid attack rate, abiotic factors

## Abstract

*Aphidius colemani* Viereck (Hymenoptera: Braconidae) is a solitary endoparasitoid used for biological control of many economically important pest aphids. Given its widespread use, a vast array of literature on this natural enemy exists. Though often highly effective for aphid suppression, the literature reveals that *A. colemani* efficacy within greenhouse production systems can be reduced by many stressors, both biotic (plants, aphid hosts, other natural enemies) and abiotic (climate and lighting). For example, effects from 3rd and 4th trophic levels (fungal-based control products, hyperparasitoids) can suddenly decimate *A. colemani* populations. But, the most chronic negative effects (reduced parasitoid foraging efficiency, fitness) seem to be from stressors at the first trophic level. Negative effects from the 1st trophic level are difficult to mediate since growers are usually constrained to particular plant varieties due to market demands. Major research gaps identified by our review include determining how plants, aphid hosts, and *A. colemani* interact to affect the net aphid population, and how production conditions such as temperature, humidity and lighting affect both the population growth rate of *A. colemani* and its target pest. Decades of research have made *A. colemani* an essential part of biological control programs in greenhouse crops. Future gains in *A. colemani* efficacy and aphid biological control will require an interdisciplinary, systems approach that considers plant production and climate effects at all trophic levels.

## 1. Introduction

*Aphidius colemani* (Hymenoptera: Braconidae) is a solitary, koinobiont endoparasitoid of aphids, and is one of the most successful commercial biological control agents used in greenhouse crops. Thought to be of Indian or Pakistani origin [1], this parasitoid wasp was first used in biological control programs in the early 1970s [2], and has been mass reared and sold commercially since 1991 [3]. *Aphidius colemani* is currently used throughout the world, and is available from multiple commercial suppliers. A description of the morphological characteristics and life cycle of this species can be found in Benelli *et al.* [3].

*Aphidius colemani* is mainly used to control the economically important aphids *Myzus persicae* Sulzer (green peach aphid) and *Aphis gossypii* Glover (melon or cotton aphid) [4,5,6]. *Myzus persicae* and *A. gossypii* are extremely polyphagous and attack a wide range of vegetable and ornamental crops grown in greenhouses such as peppers, cucumbers, tomatoes, bedding plants, foliage plants, and cut flowers. *Aphidius colemani* is an especially useful tool against pesticide resistant strains of these aphids [7], and can also attack important sub-species of *M. persicae*, such as *M. persicae nicotianae* [8]*.* Though *A. colemani* has a host range of over 41 aphid species [2], not all greenhouse aphid pests are controlled by this parasitoid. *Aphidius colemani* will sting the potato aphid, *Macrosiphum euphorbiae* (Thomas), but it is unable to complete development in this host [1]. Thus, *A. colemani* is not an effective control agent for this pest, nor for foxglove aphid [*Aulacorthum solani* (Kalthenbach)] or chrysanthemum aphid [*Macrosiphoniella sanborni* (Gillette)]. Since *A. colemani* cannot control all greenhouse pest aphids, other biological control agents are often released as part of an aphid management strategy (See [9,10]).

*Aphidius colemani* has many positive attributes that often make it one of the first biological control agents growers implement. In ideal circumstances, *A. colemani* can maintain aphid populations at levels similar to those resulting from pesticide applications [6], but is safer and less time consuming to apply [11]. *Aphidius colemani* has greater dispersal distance and searching activity within the greenhouse than some aphid predators such as the green lacewing *Chrysoperla rufilabris* (Neuroptera: Chrysopidae) [12]. When used in conjunction with the predatory dipteran *Aphidoletes aphidimyza* (Rondani) (Diptera: Cecidomyiidae) in a greenhouse trial, the majority of the aphid control was attributed to *A. colemani* [13]. Compared to three other economically important Aphidiine parasitoids (including *Aphidius matricariae*), *A. colemani* was the most effective at controlling *A. gossypii* in the greenhouse due to its higher parasitism rate on this host [14]*. Aphidius colemani* is also relatively easy to rear commercially, making it one of the more cost-effective aphid biological control agents on the market, at around $0.07 per adult, taking into account shipping costs and non-emergence [15]. Further, *A. colemani* can be reared in the greenhouse by growers on “banker plants”—Plants supporting a non-pestiferous aphid population as an alternate food source for the wasp when pest aphid levels are low [16]. This provides prophylactic aphid control by providing a constant source of wasps [15]. As an aphid specialist, *A. colemani* is also compatible with biological control programs for other greenhouse pests [17].

Despite the low cost, ease of use, and comparatively high efficacy of this parasitoid, no biological control agent is foolproof. Failures of *A. colemani* have been reported many times in the literature (e.g., [15,18,19,20]), and are an unfortunate reality for growers (Acheampong *et al.* [21]; S.E. Jandricic, personal observation [22]). For example, in the large floriculture production area of Ontario, Canada, 69% of growers currently use biological control as the main means of pest management. However, for many of these growers, incomplete aphid control with natural enemies is the primary reason their pest management programs still require pesticide use (J. Aalbers, personal communication [23]). So, what precipitates failures of a natural enemy like *A. colemani* that is generally successful?

According to ecological theory, for prey populations to be suppressed, the following model must be true:

r < aP*

where *r* = prey growth rate, *a* = attack rate per-predator per-unit-prey, and P* = predator abundance at equilibrium [24]. Thus, any factor which reduces parasitoid abundance (e.g., affects development time, survival, longevity, reproduction), attack rate (e.g., affects searching ability, flight capacity, host preference, prey defenses), or increases prey growth rate (e.g., affects aphid development time, survival, fecundity, defenses), could ultimately allow pest populations to grow. Greenhouses are managed, relatively closed environments compared to natural or other cropping systems. Yet, many ecological factors are still present that could negatively or positively affect the life history, fitness, or behavior of *A. colemani*.

**Figure 1:**
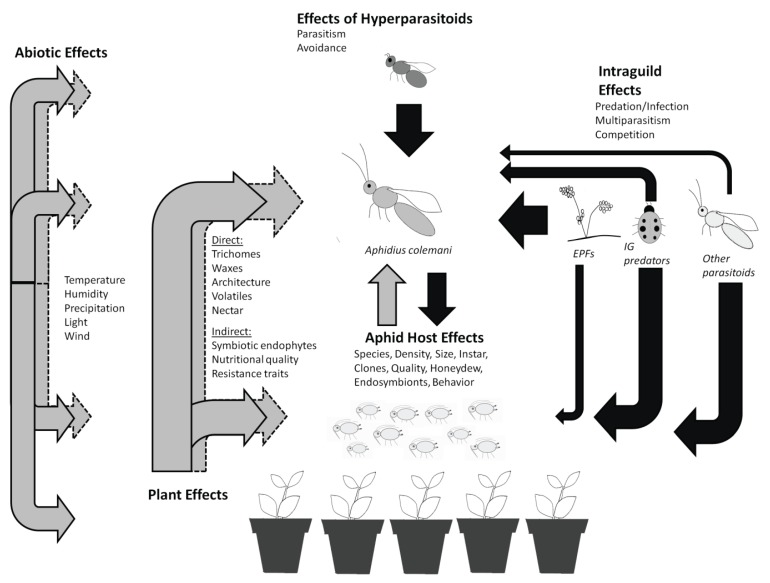
Ecological factors in greenhouse crops that can affect the efficacy of *A. colemani* and management of aphid pests. Full black arrows indicate direct negative effects on either the wasp or the pest. Full grey arrows indicate direct positive or negative effects on either the wasp or the pest. Dashed grey arrows indicate indirect positive or negative effects on either the wasp or the pest. Size of arrows approximately corresponds to size of effect, based on our literature review. EPF’s stands for entomopathogenic fungi.

Our objective was to identify the biotic and abiotic factors that may affect *A. colemani* efficacy by acting on “*a*” or “P*”. Our goal is not to review all studies relating to *A. colemani*. Instead, we focus on factors at each trophic level that could affect efficacy of *A. colemani* (Figure 1). Trophic levels include the crop plant, the host aphid, intra- and inter-guild interactions with other natural enemies, and hyperparasitoids. Abiotic factors such as temperature, humidity, air flow, and toxic chemicals in the environment can also inhibit the functioning of *A. colemani* in isolation or in concert with biotic effects. By identifying and synthesizing the complexities and interactions inherent in each trophic level and in the greenhouse environment, we hope to identify and prioritize the research topics which will improve the stability and reliability of *A. colemani* for aphid control in greenhouse crops (Table 1).

**Table 1:** List of ecological factors in greenhouse crops that can directly and indirectly affect the efficacy of *A. colemani*, with gaps in research for this species noted.

Factors Affecting *A. colemani*	Direct or Indirect	Type of Effect	Positive or Negative for Biological Control	Ways Biological Control by *A. colemani* is Negatively or Positively Affected	Example References for *A. colemani*
Plants	Direct	Morphological defenses (e.g., trichomes, spines, waxy layers)	Negative	Increase *A. colemani* grooming time, and may impede movement on plant	[25]
				Increased aphid handling time	[24]
		Non-defensive morphological traits (e.g., PGR effects on plant architecture)	Negative	Host-finding is more difficult	[26]
				May negatively affects mummy abundance, percent emergence, female parasitoid size, and sex ratio	[27]
		Volatile organic compounds (e.g., plant species cues alone)	Variable	Could affect host plant choice	[28,29,30,31,32]
		Volatile organic compounds (e.g., natal-host effects)	Positive	*A. colemani* may prefer host complex on which it is reared	[29,30]
		Resource provisioning (e.g., Flower nectar)	Positive	Can increase fecundity, percent emergence, female sex ratio, and longevity	[33]
			Negative	Could benefit pests and hyperparasitoids	NA
	Indirect	Good plant quality	Positive	Can increase fecundity, percent emergence, female sex ratio, and longevity	[33]
		Herbivore resistance traits (e.g., toxic allelochemicals)	Negative	May negatively affects life history traits	[32]
		Fertilizers	Positive	Increase percent emergence, mummy weight, male longevity and adult size	[34]
			Negative	Could benefit herbivore pests	NA
				Could decrease parasitism	NA
				Could affect plant defensive compounds, which can affect herbivores and their natural enemies	NA
		Plant symbionts (e.g., rhizobacteria)	Positive	Could increase crop vigor and resistance to pests	NA
			Negative	Could alter the volatile composition, which may make plants less attractive to *A. colemani*	NA
		Endophytes	Negative	Could affect reproductive ability of the F1 generation	NA
				Could increase development times	NA
				Could reduce female abundance	NA
		Varieties/species effects	Variable	Percent emergence may be reduced on some species, compared to other.	[35]
				Variance in female development time	[36]
				Variance in number of mummies	[36]
Aphid hosts	Direct	Aphid species	Variable	Offspring survival, female ratio, and size were lower on *R. padi* than *M. persicae, A. gossypii,* and *S. graminum*	[37]
				Using a poor quality aphid as an alternate host on a banker plant can benefit biological control of higher quality aphid hosts on crop plant	[38]
				If multiple pest aphid species are present in a greenhouse, there could be variable levels of control	NA
		Endosymbionts (e.g., *Regiella insecticola*)	Negative	Infected clones may be resistant to *A. colemani*	[39]
				Parasitoids could be equally attracted to infected and uninfected hosts, so they may waste their eggs and energy	NA
		Preference for *A. gossypii*	Positive	Good control if target pest is *A. gossypii*	[1]
			Negative	May experience reduced life history traits on *A. gossypii* compared to *M. persicae*	[37]
				May not perform well in multi-pest environment	NA
		Clones	Variable	Parasitism levels vary with clone (red clone > light green > dark green)	[40]
			Negative	Less effective against insecticide-resistance clones	[41]
		Host instar	Variable	Prefers 1st and 2nd instars of *A. gossypii* and *M. persicae* on eggplant	[41,42]
				Prefers 2nd and 3rd instars of *M. persicae* on pepper	[42]
				Prefers 4th and 5th instars of *A. glycines* on soybean	[43]
		Defensive behavior	Negative	Increase handling time and risk of injury	[44]
				Small parasitoids have narrower host range than large ones	[45]
		Host density	Positive	Density is positively correlated with foraging time and ovipositions	[4,46]
			Variable	Type II functional response at high-densities; Type III functional response at low-densities; Type II functional response at low-densities; Type III functional response at high-densities	[4]
		Honeydew production	Positive	Benefits *A. colemani* longevity	[47]
				Could help host finding	NA
	Indirect	NA	NA	NA	NA
Third and fourth trophic levels	Direct	Multiparasitism (*i.e.*, multiple parasitoids species parasitizing same host)	Negative	Other aphid parasitoids can outcompete *A. colemani* larvae	[48]
		Predators	Neutral	Does not avoid predator-infested plants	[49]
			Negative	Predators can reduce parasitoid abundance by eating the parasitized aphids	[50]
			Positive	Additive and synergistic effects from a diversity of natural enemies	[51]
		Entomopathogenic fungi	Negative	*Beauveria bassiana* can infect and kill adult *A. colemani* at high rates (>55% of the population)	[52,53]
				Can also infect parasitized aphids and reduce mummy formation and adult emergence	[54]
				*A. colemani* does not detect infected hosts, so wastes eggs/energy	[55]
			Neutral	*Verticillium lecanii* is safe for *A. colemani* in mummy form (5 days post-parasitism)	[54]
		Hyperparasitoids (e.g., *Alloxysta victrix and Dendrocerus aphidum)*	Negative	Parasitize *A. colemani*	[56,57]
				In the summer, when hyperparasitoid population is high, aphid control can fail	[14]
				Can affect parasitoid population on banker plants	[20]
	Indirect	NA	NA	NA	NA
Abiotic factors in greenhouses	Direct	Pesticides	Negative	Can lead to direct mortality of *A. colemani*	[58,59,60,61,62]
		Temperature	Variable	Temperatures could exceed development threshold for *A. colemani* (e.g., larvae generally cease development at 30 or 31 ºC)	[63,64]
				Development is roughly fastest between 22 °C and 28 °C	[17,64,65]
				Faster development can result in smaller parasitoids, with shorter lifespans and reduced fecundity	[66]
				Can develop at temperatures as low as 10 °C	[63,65]
				Elevated temperature can increase parasitoid performance	NA
		Dynamic climate regimes	Variable	Dynamic climate regimes could affect efficacy	NA
		Humidity	Variable	Could affect fecundity, hatching and predation	NA
				Could affect flight and dispersal	NA
				Parasitoid eclosion and adult longevity could decrease at high humidity levels	NA
				Low humidity levels could have negligible effects on foraging	NA
		“Precipitation”	Negative	Could reduce foraging and increase parasitoid cleaning time	NA
		Light (e.g., light emitting diodes (LED), photoselective screens (e.g., UV absorbing), and changes in photoperiod)	Neutral	Reduced UV light has no effects on *A. colemani* performance	[67]
		Wind	Negative	Could reduce oviposition and increase resting behavior of parasitoid	NA
	Indirect	Pesticides (including residual effects)	Negative	Can be exposed to insecticides even through honeydew and nectaries	[59,68,69]
				Could experience decreased attraction to aphids on treated plants	NA
				Reduced re-invasion of areas treated with pesticides	[70]
				Could cause a reduction in foraging behavior	NA
				Can reduce oviposition and fecundity	[59,62,71]
				Could impact development time and sex ratio	NA
		Temperature	Negative	Can increase *A. gossypii* populations	[72]
			Variable	Populations of *A. gossypii* and *M. persicae* can still increase at 30 ºC–33 ºC	[67,73]
		Light (e.g., light emitting diodes (LED), photoselective screens (e.g., UV absorbing), and changes in photoperiod)	Variable	Changes in lighting can alter plant nutritional quality, physical or chemical defenses, and/or volatile emissions or profiles, which in turn could affect *A. colemani*	NA
				Reduction of UV light does not negatively affect performance of *A. colemani*	[74]
		Wind	Negative	Could interfere with male mating flights	NA

## 2. Plant Effects on Pest Suppression by *A. colemani*

### 2.1. Direct Plant Effects

Morphological characteristics of plants, such as trichomes, spines, and waxy layers can deter herbivore colonization and feeding [75]. Such morphological defenses can also reduce biological control by reducing natural enemy colonization and foraging efficiency [25,75,76,77,78,79]. Desneux and Ramirez-Romero [25] compared *A. colemani* efficiency at attacking *Myzus persicae* (Hemiptera: Aphididae) on *Brassica napus* with and without epicuticular wax. *Aphidius colemani* spent 20% more time grooming and had 15% more failed stings on waxy plants than on the non-waxy *B. napus* [25]. Similarly, glandular trichomes caused *A. colemani* to spend more time searching, leading to nearly ten times fewer parasitized aphids on potato species with glandular trichomes than on the species without [80]. Most of the aphid control was actually due to the effects of trichomes, with only 5.6% attributed to parasitism [80]. And, parasitoid mortality was nearly three times higher when trichomes were present than when absent [80]. Determining the release rate that provides effective control on plants with more challenging morphological characters, would allow growers to minimize their costs, while maximizing aphid control.

Other morphological traits such as leaf size, leaf texture, and number of branches, which have not necessarily evolved as plant defenses, can also affect biological control. While these traits have not been studied for *A. colemani*, these architectural characteristics have been shown to impede foraging efficiency of other parasitoids and natural enemies [76,81,82,83,84]*.* Growers also actively alter plant architecture with horticultural techniques, including pruning [85] and by applying plant growth regulators (PGRs)—organic compounds used to modify plant growth and/or development [27]. The effect of PGRs on *A. colemani* has been studied, and Paclobutrazol, commonly used to create more compact, bushier plants, was shown to reduce *A. colemani* foraging efficiency by providing aphids with more concealed feeding locations than the sparser, untreated plants [26]. PGRs can also reduce *A. colemani* mummy abundance, percent emergence, female parasitoid size, and sex ratio [27]. In fact, no adults emerged from mummies on plants treated with ancymidol, a PGR used to reduce internode elongation. The mechanisms of how many PGRs affect parasitoid fitness are unclear but the direct and indirect effects of these compounds should be considered when designing or evaluating a biological control program. Whether plant architectural differences are grower-induced or due to natural variation, it is important for growers to understand the effects these changes may have on biological control outcomes. Plant architecture variation could explain potential failures in biological control between seasons and years and between plant species/cultivars.

Morphology is not the only plant attribute that can directly affect *A. colemani*. Plants also release volatile organic compounds, which are used by natural enemies to track prey [86]. *Aphidius colemani* searching efficiency is highly dependent on these volatiles, and can vary with the aphid-plant species combination [29,30,31,32,56]. The host-plant species’ chemical cues, alone, can also influence host selection [29,32]. For example, when given the choice between the odor of uninfested rape (*Brassica napus*) leaves and uninfested Chinese cabbage (*Brassica rapa ssp. chinensis*) leaves, *A. colemani* showed a preference for the rape, regardless of the plant on which they had been reared [29]. Thus, *A. colemani* foraging efficiency is very likely to vary across greenhouse crops due to differences in plant chemical cues and defensive volatile profiles.

Plant derived cues can also affect parasitoid host choice via natal-host effects. Using a Y-tube olfactometer, Storeck *et al.* [29] found that *A. colemani* showed a preference for the host-plant complex on which it was reared. Douloumpaka and van Emden [30] found that *A. colemani* emerging from aphids on plants preferred the odor of that plant species, whereas those emerging from aphids reared on artificial diet showed no preference. Additionally, parasitoids can be imprinted with the odor of plants that are near the one on which they were reared [87]. *Aphidius rhopalosiphi* emerging from aphids on wheat grown near tomato were imprinted with the tomato volatiles, and subsequently preferred the odor of wheat grown near tomatoes [87]. Given the observations by Douloumpaka and Van Emden [30], similar behavior can be expected for *A. colemani.* Though foraging experience can override this conditioned preference [29], it may still be helpful to position *A. colemani* banker plants near the target crop to ensure attraction to the target area.

Adult parasitoids often consume flower nectar for carbohydrates and other nutrients [47]. Such resource provisioning by plants can benefit parasitoid life history traits [33,88,89] and parasitism [88,90]. *Aphidius colemani* feeding on nectar from the shrub *Photinia x fraseri* Dress (Rosaceae) had higher fecundity, percent emergence, female sex ratio, and longevity when compared to a blank control [33]. However, nectar sources may also work against biological control programs by benefiting pests [91,92,93,94] and hyperparasitoids [89]. Although longevity of *Aphidius ervi* (Haliday) (Hymenoptera: Braconidae) increased in the presence of flowering buckwheat, *Fagopyrum esculentum* (Moench), longevity of its hyperparasitoid *Dendrocerus aphidum* (Rondani) (Hymenoptera: Megaspilidae) also increased, causing it to live 2.5 to 3 times longer than *A. ervi* [89]. Thus, resource provisioning could indirectly increase aphid abundance. Pest aphid populations can also directly benefit from resource provisioning. When oats were interplanted with fava beans, *R. padi* population densities nearly doubled compared to oat monocrops [91]. In this case, parasitism was inversely density dependent, and parasitoids were not able to keep up with the growing aphid population. But, in other cases, an increase in pest abundance can be accompanied by an increase in parasitism (See [93]). Consequently, the outcome of aphid biological control programs using *A. colemani* is likely to vary in effectiveness over time, as plants flower. Effectiveness is also likely to vary between greenhouses containing flowering plants and those with vegetative plants only. Greater research in this area is needed to determine the greenhouse crop type (flowering *vs.* non-flowering crops, mixtures) in which *A. colemani* is likely to function optimally.

### 2.2. Indirect Plant Effects

Plants can indirectly affect parasitoids via their quality (Figure 1). Plants of low quality, due to low nutritional value or high plant defenses, can reduce the size, quality, or fecundity of aphid hosts. Thus, fewer or lower quality aphid hosts may be present for parasitoid reproduction [75,95]. The reverse is also true: High quality plants and resources can improve *A. colemani* life history traits, making them potentially better biological control agents [33]. However, it is often difficult to separate effects of host plant quality from the effects of aphid species or clone quality on parasitoid fitness. Thus, summation of indirect plant effects on *A. colemani* can be complicated. With this in mind, in this section, we isolated host-plant effects by highlighting papers that manipulated plant species or cultivar, while keeping the aphid species constant.

Production of toxic allelochemicals is one way plants reduce the survival and reproduction of herbivore pests [96,97]. In agricultural crops, such chemical resistance traits are often increased through plant breeding [98] and their effects on herbivores are relatively well understood [95]. Comparatively few studies have assessed the effects of herbivore resistant plants on *A. colemani* or other natural enemies. Kalule and Wright [32] compared the effects of three common cabbage cultivars with varying levels of resistance to the aphids *Brevicoryne brassicae* L. and *M. persicae* on *A. colemani* life history traits. *Aphidius colemani* females emerging from aphids reared on highly resistant cabbage cultivars had reduced adult longevity, though no other fitness characteristics were affected [32]. Other possible negative effects of plant allelochemicals on *A. colemani*, such as reduced clutch size, longevity and parasitism rates, have to be surmised from other non-aphid parasitoid species [99,100]. For example, longevity of Mexican bean beetle parasitoid larvae [*Pediobius foveolatus* (Hymenoptera: Eulophidae)] was negatively affected by herbivore resistance in soybeans, and was lowest on the cultivars most resistant to Mexican bean beetle hosts [*Epilachna varivestis* (Coleoptera: Coccinellidae)] [99]. If parasitoids are less likely to parasitize hosts on toxic plants, and have reduced abundance via decreased longevity and clutch size, they are unlikely to be effective biological control agents. This may not be a cause for concern in ornamental crop production, where varieties are not usually bred for herbivore resistance, but this is important in greenhouse vegetable crops such as tomato, pepper, and lettuce.

Fertilizers can improve plant quality for herbivores and subsequently affect the hosts’ suitability for parasitoids [101]. Again, although the effects of fertilizers on herbivores have been well studied [102], little is known about their effects on parasitoids. Aqueel *et al.* [34] found that parasitism, percent emergence, mummy weight, male adult longevity, and *A. colemani* size were increased by adding nitrogen fertilizer. Other fitness indicators, such as sex ratio, were unaffected. Although this study showed positive effects of nitrogen on *A. colemani*, nitrogen can also increase pest hyperparasitoid population growth [103,104,105], which may lead to negative effects on biological control programs. The type of fertilizer used can also affect parasitism, as was shown by decreased parasitism of the aphid *Brevicoryne brassicae* on cabbage plants fertilized with organic chicken manure compared to those using synthetic fertilizer [106]. Additionally, fertilizers affect plant defensive compounds, including glucosinolates [107,108], which have the potential to affect herbivores and their natural enemies [109]. Fertilizers should therefore only be used to the extent that they improve plant growth, yield, or aesthetics, as excess fertilizer may cause more harm than good [110,111].

Plant symbionts such as rhizobacteria can affect interactions between herbivores and their natural enemies [112], by increasing crop vigor and potentially increasing plant tolerance to pests ([113] but see [114]). Initial studies on parasitoids showed that rhizobacteria can alter the volatile composition of plants by interfering with the jasmonic-acid pathway [115]. Thus, rhizobacteria-colonized plants infested with *M. persicae* were less attractive to the parasitoids due to a breakdown in defensive chemical signaling [115]. Zytynska *et al.* [116] demonstrated a significant variation in size of *A. rhopalosiphi* when reared on aphids feeding on barley plants infected with rhizobacteria. However, the variation in parasitoid size was affected by both plant genotype and the aphid host, demonstrating the complexity of interactions that plant symbionts can have with the 3rd trophic level. A study assessing the effect of rhizobacteria on parasitism by *A. colemani* using a single aphid and plant species could inform biological control program recommendations.

Endophytes are symbiotic fungi which can protect plants from herbivores by producing toxic alkaloids in exchange for nutrition from the plant [117]. The effects of endophytes on herbivores have been studied in many systems, but their effects on natural enemies are relatively unknown [112]. No study, to date, has investigated the effects of endophytes on *A. colemani*. However, Härri *et al.* [112,118] investigated the effects of endophytes on *A. ervi* reared on a grain aphid. They found that plants infected with the endophyte *Neotyphodium lolii* negatively affected the reproductive ability of the F1 generation of *A. ervi*, and fewer mummies were produced [112]. Understanding endophyte effects on aphid and parasitoid population dynamics may help explain failures of *A. colemani* in particular crops and will improve biological control recommendations. For example, for crops with high endophyte populations, release rates and natural enemy composition may need to be modified to reduce aphids below threshold abundance.

The above examples detail cases where plants indirectly affect *A. colemani* through a known mechanism. However, there are also many cases where the mechanisms responsible are unknown or not yet identified. Jandricic *et al.* [35] observed a lower percent emergence of *A. colemani* reared on *R. padi* on oats (*Avena sativa* L.) compared to those reared on barley (*Hordeum vulgare* L.), rye (*Secale cereal* L.) or wheat (*Triticum aestivum* L.). The number of parasitoids available for biological control was therefore affected by plant species, though the reasons for this are unclear. Rearing *A. colemani* on different cultivars/varieties can also affect parasitoid fitness [36]. Plant genotype can cause a variance of almost 10% in female *A. colemani* development time, and 14% in number of mummies produced [36]. Studies like these provide information that can help growers select plants to optimize *A. colemani* production, and should be continued in the future. Such studies also draw attention to the unintended effects of plant breeding programs on the efficacy of biological control. While identifying the mechanisms by which the plants may be affecting the parasitoid is important, we understand that in many cases, the mechanisms may be difficult to tease apart, due to the indirect interactions between plants and natural enemies (see [98]).

## 3. Host Aphid Effects on Pest Suppression by *A. colemani*

The larvae of koinobiont parasitoids are intimately associated with their host [119] (Figure 1). Therefore, distribution, abundance, and performance of *A. colemani* in the greenhouse will depend on the quality of their host aphid. Parasitism is generally restricted to a comparatively (to predators) narrow range of species which the parasitoid has the behavioral and morphological adaptations to locate and successfully attack. Within this host range, characteristics such as instar, body size, color morph, and colony density affect pre-and post-parasitism success, as well as prey preference [120] (Table 1). Impacts of these factors on the success of *A. colemani* as an aphid biological control agent are detailed below.

### 3.1. Host Aphid Effects on Parasitoid Development and Fitness

Although *A. colemani* can reproduce in over 41 aphid host species [2], it is primarily used as a biological control agent for *M. persicae* and *A. gosypii*, two of the four ubiquitous pest species in greenhouses. *Aphidius colemani* life history traits can differ by aphid host [37], which may affect its population size and ability to suppress pests. A well-studied example of how aphid host affects wasp fitness is with *Rhopalosiphum padi* compared to other aphids. Despite its common use in banker plant systems for *A. colemani*, several studies have shown that *R. padi* is actually a relatively poor host for this parasitoid [5,37,38]. Parasitoid offspring survival, proportion of female offspring, and size were significantly lower on *R. padi* than *M. persicae*, *A. gossypii*, and *S. graminum* [37]. Similarly, Bilu *et al.* [5] suggested that *R. padi* was the least suitable host for *A. colemani* compared to *A. gossypii* and *M. persicae*, as determined by offspring body size. Reduced female offspring size and offspring survival was also found by Prado and Frank [38] when using *R. padi* compared to *M. persicae* as host*.* Smaller parasitoids can carry fewer eggs [121], may be less efficient at host searching [122], and will fly shorter distances than larger parasitoids [123]. Taken together, these effects make it seem like *R. padi* is an extremely poor choice for banker plant systems in greenhouses, however, in this case, relatively lower fitness on the alternate host is a benefit. This is because *A. colemani* females tend to leave *R. padi* on the banker plants to forage for better hosts (e.g., pest aphids) on the crop plants [38]. While a number of recent studies have investigated the use of banker plants at suppressing a single pest species (e.g., van Driesche *et al.* [15]; Prado and Frank [38]), using banker plant systems to suppress multiple pest aphids in a greenhouse is yet to be investigated. This could be especially important given that studies with other aphid biological control agents have suggested that prey preferences can lead to differential control in the greenhouse [124].

*Aphidius colemani* fitness can also be affected by aphid clonal lines within the same species—an important consideration given that aphids reproduce parthenogenetically in greenhouses. A few key studies have suggested that different secondary endosymbionts in aphids can confer resistance to parasitoid attacks by causing larval mortality of developing parasitoids [125,126]. However, to our knowledge, only one study has investigated the effects of aphid endosymbionts on *A. colemani* parasitism. Clones of *M. persicae* and *Aphis fabae* infected with the endosymbiont *Regiella insecticola* were strongly resistant to *A. colemani*, as indicated by lower number of mummies [39]. When presented with aphids with and without such endosymbionts, the closely related species *A. ervi* was equally attracted to both, suggesting that parasitoids may be wasting eggs and energy parasitizing resistant aphids [125]. Larval mortality, and wasted energy and eggs can reduce parasitoid abundance and efficacy. Although a logical alternative to parasitoids in this scenario would be predators, consumption of aphid secondary symbionts can reduce predator survival as well [127,128]. Thus, if growers notice a lack of efficacy of biological control agents for an emerging aphid population, the presence of resistance-conferring endosymbionts may be to blame, and pesticides may have to be used (but see Section 5). Currently, studies are investigating the use of antibiotics to eliminate aphid endosymbionts as a means of improving aphid control [129].

### 3.2. Host Effects on Aphid Acceptance and Suppression by A. colemani

Biological control outcomes for aphids can be significantly affected by prey preference of the natural enemy involved (e.g., Bergeson and Messina [130]). Although *A. colemani* parasitizes many aphid species, it seems to have an apparent genetic bias in attack performance, leading it to attack *A. gossypii* more readily than *M. persicae* [1]. This bias was strong enough to cause *A. colemani* which had been reared on *M. persicae* for multiple generations to switch hosts when given the option [1]. Similar observations made by Sampaio *et al.* [131] and Bueno *et al.* [64], and anecdotal observations made by biological control specialists in Canada confirm that *A. colemani* seems to better control populations of *A. gossypii* than *M. persicae* (G. Murphy, personal communication [132]). There is no simple explanation for this apparent preference, however. No fitness benefits for *A. colemani* emerging from *A. gossypii* have been noted (but see [133]—potential benefit for male mating ability), and in fact, female size for *A. colemani* is smaller when emerging from *A. gossypii* compared to *M. persicae* [37]*.* As Benelli *et al.* [3] suggest, however, preference may not be precisely matched with host quality variables. Further studies are needed to clarify both preference and performance of this parasitoid on greenhouse aphid pests, especially if it is being marketed to growers as an effective solution for both *M. persicae* and *A. gossypii*. Additionally, studies should determine how *A. colemani* reacts in the presence of suitable (e.g., crops infested with both *M. persicae* and *A. gossypii*) and/or unsuitable aphid host species, as studies with other aphid biological control agents in greenhouse crops have suggested that such multi-pest environments can lead to differential control [134,135].

Parasitoids use aphid physical, chemical, and behavioral attributes to select a host [120,136]. Thus, variation between aphid clonal lines within a same species can influence aphid acceptance and suppression by *A. colemani* [40,120,137]*.* For example, *A. colemani* performance varied when presented with three *M. persicae* clones (light green, dark green and red) collected from greenhouse pepper plants [40]. *Aphidius colemani* parasitized more red clones than light green and dark green clones, with dark green clones least parasitized. When assessing the long-term (4-week) effects of these interactions on aphid suppression, Gillespie *et al.* [40] found that parasitism reached 100% in cages with the red and light green clones, but only about 50% of the dark green clones. The authors suggest that the differences in aphid suppression are related to the aphids’ life history traits. Dark green clones (which lacked expression of a particular esterase involved in insecticide resistance) had a higher reproductive rate, and thus may have been least vulnerable to suppression by *A. colemani* [40]. Different clones of *M. persicae* have also been associated with different levels of insecticide resistance [137,138], and *A. colemani* is less effective against certain insecticide-resistant clones [138]. This reduced control could be due to differential survival and development rates in the insecticide-resistant aphids [138]. Though this may be worrisome for growers, insecticide-resistant clones may have fitness tradeoffs including reduced reproductive rates, and reduced response to alarm pheromones, which could lead to selection against these clones in the absence of insecticides [138]. Furthermore, combining aphid predators, such as coccinellid species, with kaolin (an insect repellent) have shown promising results for the control of insecticide-resistant *M. persicae* [139]. Altogether, this provides an argument for relying on biological control as the primary means of aphid control. Introducing pesticides can interact with both aphids and their natural enemies in unexpected ways, and complicate biological control-based integrated pest management (IPM) programs for other pests in the same system.

Differences between aphid instars can also affect *A. colemani* efficacy [43,140]. In terms of pest control, the theoretical ideal would be for *A. colemani* to prefer 1st and 2nd instars, as this would kill pest aphids before they reproduced. If later instars (3rd or 4th) are attacked, both *M. persicae* and *A. gossypii* are able to reach adulthood and produce a limited number of offspring before becoming mummies [41]. Similarly, Lin and Ives [43] noted that although they had reduced fecundity, later-instar *Aphis glycines* were able to reproduce after reaching adulthood for up to three days following parasitism. Overall, results from studies on the actual instar preference of *A. colemani* are widely variable. This suggests that *A. colemani* preference for specific instars varies with aphid species and/or host plants. For instance, Perdikis *et al.* [41] showed that *A. colemani* preferred to parasitize 1st and 2nd instars of *A. gossypii* and *M. persicae* over the older, larger hosts. Martinou and Wright [42], however, observed a preference for intermediate instars (2nd and 3rd) of *M. persicae* reared on pepper (*Capsicum annuum* L.), with the preference shifting to 1st–3rd instars when *M. persicae* was reared on aubergine. Such differences in instar preference may be due to differences in instar size between species [121], parasitoid size, host defensive behaviors, and immune responses [141].

Older and larger aphids can better defend themselves from parasitoids [142]. These defenses, which include kicking, dropping, shaking their body, and running away [142] can increase parasitoid handling time and risk of injury [44,143,144,145]. As parasitoids alter their host-selection behavior in relation to their own body size, large and small parasitoids of the same species prefer larger and smaller aphids, respectively [144,146]. The inverse relationship between parasitoid size and handling time means that smaller aphid hosts are more often parasitized by smaller *Aphidius* individuals (because they have not yet developed effective defenses; [144]), while larger *A. colemani* have a wider range of accepted instars [44,45]. A strict preference for specific instars could affect the potential of the parasitoid to exploit aphid populations that differ in size structure. Thus, *A. colemani* ability to parasitize a wide range of host instars may be beneficial in biological control in greenhouses, where multiple crops and aphids combinations co-occur.

### 3.3. Host Density Effects on Aphid Suppression by A. colemani

Aphid density can influence parasitoid searching time and number of patch visits [4,46]. Stadler and Volkl [46] found that density of the aphid *Pentalonia nigronervosa* Coq. (Hemiptera: Aphididae) was positively correlated with the amount of time *A. colemani* spent foraging, which, in turn, was positively correlated with number of ovipositions [46]. *Aphidius colemani* showed similar behavior in a different study with *A. gossypii*, suggesting this is independent of aphid species [4]. In this study, *A. colemani* also arrived earlier and searched longer on heavily infested than lightly infested leaves, but did not always discover hosts on low-density leaves [4]. This led the authors to suggest that *A. colemani* exhibits a type III functional response on low-density patches (linear increase in parasitism with increasing host density, until a maximum is reached), while exhibiting a type II functional response (decreasing parasitism with increasing host density) at higher aphid densities. However, the converse was found by Byeon *et al.* [147]. Differences in these findings could be explained by different leaf sizes or aphid species used. A more consistent comparison of *A. colemani* functional response on different aphid species on the same sized leaf could provide insight into how well *A. colemani* will react to differentially infested plants. More importantly, it may provide a clearer picture of the efficacy of *A. colemani* at low densities. Currently, *A. colemani* is often recommended for aphid control at low densities simply because it is cheap [51], rather than because of its effectiveness.

Honeydew production by aphids could also explain some of the differences in *A. colemani* response to varying aphid densities, as *Aphidius* wasps are known to use honeydew as contact kairomones to locate host aphids [148]. The presence of honeydew can increase the amount of time *Aphidius nigripes* (Hymenoptera: Braconidae) dedicates to searching for aphids, and host-searching is focused closest to the honeydew [148]. As honeydew concentration increases with aphid densities, it is likely that *A. colemani* will focus initial efforts on highly infested plants, rather than plants with few aphids. Honeydew, like flower nectar, can also provide non-prey food for parasitoids [47]. Host species can affect honeydew quality for *A. colemani*, which can affect its longevity [47]. *Aphidius colemani* lived nearly 9 days longer when feeding on honeydew produced by *M. persicae* than it did when feeding on honeydew produced by *B. brassicae* on the same plant [47]. Aside from investigating the life history benefits of honeydew on parasitoids, few studies have actually assessed the impact of honeydew on biological control [47]. Further research is needed to determine if, and how honeydew affects the outcome of biological control.

## 4. Considerations at the 3rd and 4th Trophic Levels: Effects of Competition, Intraguild Predation, Hyperparasitism and Multiparasitism

Interactions between *A. colemani* and other organisms at the 3rd trophic level have the potential to affect aphid control outcomes (Figure 1; Table 1). One consideration is competition with other parasitoid species for hosts. Parasitoid abundance can be reduced through direct competition for prey, or multiparasitism (multiple parasitoid species laying eggs in the same host). In the latter case, one larva usually outcompetes the other (e.g., [149,150]) as in the case of *A. colemani* and *Lysiphlebus testaceipes* (Hymenoptera: Aphididae) (also commercially available), where *L. testaceipes* usually “wins” [48]. Sampaio *et al.* [48] suggest this may result in the displacement of *A. colemani* if the two are used together for *A. gossypii* control at low aphid densities. However, displacement of *A. colemani* by another parasitoid species has yet to be shown in a greenhouse study. Overall, displacement is unlikely to be a concern for growers using high, weekly releases of various parasitoid species. However, it may become an issue if banker plants are used as the main source of wasps, as there have been anecdotal reports of *A. ervi* taking over banker plants intended for the open rearing of *A. colemani* [124]. This would likely go unnoticed by the grower and outbreaks of *M. persicae* and *A.gossypii* may follow.

As concerns have recently been raised that *A. colemani* shipments might actually be a mix of closely-related species, analyses of species competition within these populations is an important avenue for future research. Tomanović *et al.* [151] used morphological and genetic analysis to reveal that species previously synonymized with *A. colemani* are likely separate species. Thus, parasitoids sold as “*A. colemani*” may actually be a complex of *A. colemani*, *A. platensis*, and *A. transcaspicus* (though work by Frewin *et al.* [152] does not support this). If some commercial suppliers have contamination with multiple parasitoid species, there is likely to be subtle physiological or behavioral differences between these species, as well as host range differences [151]. Thus, aphid control by commercial populations of this natural enemy may be affected by the ratio of one species to another. Further, confirmation of the identity and characteristics of commercial populations of *A. colemani* is needed to limit any unpredictability in control that may result from mixed-species populations.

*Aphidius colemani* also interacts with predators in the greenhouse. These can be predators which are purposefully released for aphid control (e.g., lacewings, ladybird beetles, predatory bugs like *Dicyphus* spp.) or naturally occurring (e.g., syrphid flies). Many predators readily consume aphids that have already been parasitized by Aphidiine wasps (e.g., [50,153,154,155]) obviously reducing parasitoid abundance. Though *A. colemani* does not appear to avoid predator-infested plants like some parasitoid species [49,86], predator cues may somewhat reduce parasitism rates by *Aphidius* species [50,156]. Despite these effects, few field-crop studies have demonstrated disruption of biological control when a generalist predator and aphid parasitoid co-occur (see [157,158]). Far mo re agricultural studies have revealed positive effects of natural enemy diversity on aphid control (e.g., [154,159,160,161,162,163]). This is thought to be due largely to both additive and synergistic effects from a diversity of natural enemies [51,154]. This positive trend appears to hold up the greenhouse. Snyder *et al.* [164] and Bilu and Coll [50] both show that the use of a coccinellid predator with an aphid parasitoid improved aphid control over time. Messelink *et al.* [51] demonstrate improved aphid control with *A. colemani* and the addition of the generalist predatory bug *Orius majusculus* in sweet pepper. The combination of predators with *A. colemani* may be especially useful when multiple aphid species are present in the greenhouse (see Section 3.1), when other soft-bodied greenhouse pests co-occur (see [165], or on complex plant species [26,27]).

The combination of *A. colemani* with entomopathogenic fungi for aphid biological control can be problematic. The most popular commercially-available fungal products, based on strains of *Beauveria bassiana* (Balsamo) Vuillemin*,* can infect and kill adult *A. colemani* at high rates (>55% of the population) in the lab [52] and the greenhouse [53]. This fungus can also infect already parasitized aphids, reducing mummy formation and adult parasitoid emergence by up to 83% in closely related wasp species [166]. Similar results have been seen with other fungal species, such as *Verticillium lecani* (Zimm.), where *A. colemani* emergence was reduced by 90%–100% [54]. Intra-guild interactions between parasitoids and fungi are asymmetrical, and the “winner” is determined by timing of oviposition/infection [167]. Generally, the fungi will outcompete parasitoids unless wasp oviposition takes place at least 4 days before infection [54,166,168]—meaning sprays of entomopathogens are generally only compatible with *A. colemani* when it is in the mummy form [54]. Further, *Aphidius* species cannot readily detect entomopathogenic fungi in an aphid host, only rejecting an infected aphid if it is sporulating [55].

Currently-available fungal products, with the exception of *Verticillium* species (not yet available in North America as of 2015) generally have low pathogenicity against the top greenhouse pest aphids (see [169]). Given the side-effects on parasitoids, the use of current fungal-based products should be avoided in IPM programs for aphid control. However, the reality in the greenhouse environment is that fungal products will regularly be present, as they are highly effective for other greenhouse pests such as whiteflies (Aleyrodidae) and thrips (Thysanoptera) [170,171]. As much as possible, growers need to consider their entire biological control program, even when applying reduced-risk pesticides. Thus, timing of the application of entomopathogenic fungi should be made to ensure most of the *A. colemani* population is in mummy form [172] or re-releases of this parasitoid post-spray will be a likely necessity.

Hyperparasites are parasites that develop on or in another parasite, killing it in the process. A variety of hymenopterous hyperparasites—known as hyperparasitoids—attack *Aphidius* species and can considerably reduce their numbers. Studies have shown significant non-consumptive effects of hyperparasitoids on parasitic wasps, including deterrence of *Aphidius* foraging in patches with hyperparasitoid volatiles (e.g., Holler *et al.* [173] and Petersen *et al.* [174]). The most common hyperparasitoid species of *A. colemani* include *Alloxysta victrix* (Westwood) (Hymenoptera: Cynipidae), the most common hyperparasitoid of *A. colemani* in greenhouse pepper crops in England [56], *Dendrocerus aphidum* (Hymenoptera: Megaspilidae), the most abundant species in pepper greenhouses in the Netherlands [57], and *Dendrocerus carpenteri*, the dominant hyperparasitoid in pepper greenhouses in British Columbia, Canada [21]. A plethora of other hyperparasitoid species are also reported from around the world [14,57,175,176]. The effects of hyperparasitoids on biological control in greenhouses are not well studied, but it is thought that some hyperparasitoid species may be more successful in the greenhouse than the field due to temperature differences [21]. Additionally, general trends in hyperparasitoid density based on growing seasons are suggested in the literature. van Steenis [14] found that hyperparasitism did not interrupt aphid control in the spring in the Netherlands, when hyperparasitoid density was relatively low. However, in the summer, the ratio of hyperparasitoids to *A. colemani* was much higher, and aphid control failed. A similar study from Japan showed a high rate of hyperparasitism on *A. colemani* banker plants in late spring, across 4 years of study in commercial greenhouses [20]. In some years, hyperparasitism rates reached almost 100% on banker plants by June. Acheampong *et al.* [21] found that hyperparasitism rates generally peaked from June-August in British Columbia, Canada. Together, these studies raise concerns over the efficacy of releases of *A. colemani* in summer months especially, where high rates of aphid population increase, temperatures unfavorable to *A. colemani* (*i.e.*, ≥30 °C), and higher numbers of hyperparasitoids may act together to derail aphid control [14,63,177]. However, some operations (especially organic growers) may observe hyperparsitoids disrupting aphid biocontrol as early as the spring if the hyperparasitoids are able to overwinter in the greenhouse [57].

The reliability of *A. colemani* banker plants as a biological control strategy is also affected by hyperparasitoids [20]. Hyperparasitism may be largely responsible for the 30% failure rate of banker plants to control aphids, as reported by growers in Japan [20]. It is likely that long-term use of the same banker plants by Nagasaka *et al.* [20] (who only re-seeded every 3–4 months) contributed to high levels of hyperparasitoids over time due to their longevity (*ca.* 4–6 months for adults of some hyperparasitoid species; [178]). For best performance, we recommend the replacement of banker plants every 3–4 weeks, to ensure healthy populations of banker-plant aphids [35], reduce the incidence of mildew like molds [35,179], limit space taken up by banker plants (T.J. McClure and S.D. Frank, unpublished data [180]) and remove reservoirs of hyperparasitoids. However, replacement rates may need to be even higher in summer months. Some IPM consultants in Ontario, Canada have found that the only way to prevent aphid problems from June to August is to replace banker plants every 2.5 weeks (M. Short, personal communication [181]), or even remove them entirely during this time-period and rely on pesticide sprays for aphid control. Further studies are needed to determine: (i) the timing of hyperparasitoid infestations in different regions; (ii) how different maintenance schemes for banker plants can potentially mediate risk from these organisms; (iii) if there are ways to provide parasitoids with refuges from hyperparasitoids within the greenhouse; and (iv) if the addition of another biological control agent during the summer can stabilize aphid control by *A. colemani* in the face of hyperparasitism.

## 5. Abiotic Considerations

Many abiotic factors are present in greenhouses that can act alone or interact with biotic factors to affect biological control programs (Figure 1; Table 1). Pesticides are one of the most important abiotic factors in agricultural systems. Non-target effects of pesticides on *A. colemani* have been well studied in terms of direct contact activity (e.g., [58,59,60,61,62]), and there is a clear need to evaluate novel chemicals as they come on the market. Residual toxicity of pesticides (*i.e.*, the period after application when they still pose a threat) on *A. colemani* is also recognized as an important factor (e.g., [59,68,69]), with most growers understanding that chemicals with shorter residual times are generally safer for natural enemies (S.E. Jandricic, personal observation [22]). However, *A. colemani* is more susceptible to insecticide residues on the foliage than many other natural enemies used in greenhouse biological control [68], and can continue to be exposed to systemic insecticides through aphid hosts, honeydew and nectaries [182]. Thus, greenhouse IPM programs for aphids would benefit from research to specify safe release intervals following application of different pesticides for *A. colemani.*

Open-access databases summarizing information of contact effects on *A. colemani* and persistence of chemicals can be found at http://www.biobest.be/neveneffecten/3/none/ and http://side-effects.koppert.nl/. However, these databases should not be used to find pesticides to regularly spray in conjunction with *Aphidius*. Rather, they should serve as a guide when selecting pesticides to use in “hot spots” only (smaller areas of aphid outbreaks), in order to provide *A. colemani* with refugia from pesticide effects. This is important, because even pesticides classified as “harmless” by compatibility databases have the potential to cause up to 25% mortality of *A. colemani* populations. “Slightly harmful” pesticides can cause up to 50% mortality. Though these categories concur with IOBC guidelines for pesticide compatibility with natural enemies [183], it is likely that these mortality levels represent a significant loss of protection against aphids, especially since natural enemy releases at effective, continuous rates are key to aphid biological control [10]. Similarly, pesticides can have important “indirect” effects (*i.e.*, sub-lethal or latent effects [71]) on natural enemies (see [184]), but these have not yet been clearly factored into open-access databases. Indirect effects of pesticides on *A. colemani* and other *Aphidius* spp. include decreased attraction to aphids on treated plants and re-invasion of insecticide treated areas, reduced foraging, fecundity, oviposition, increased development time and a strongly male-biased sex ratio [59,62,70,185,186,187,188,189]. Private databases (e.g., ipm-impact.com) contain some of this information, but their subscription costs are likely prohibitive to growers and smaller IPM consulting companies.

*Aphidius colemani* populations are heavily influenced by temperature, since this directly affects wasp development time (time from egg to adult emergence). Though reports of optimal development temperatures for *A. colemani* vary in the literature [17,63,64,65], development is roughly fastest between 22 °C and 28 °C. Variations in populations are likely due to host plant effects (as demonstrated by Zemek *et al.* [190], physiological differences in different host aphids at different temperatures, biotype effects, or even variation in wasp source population [191].

At extremely high temperatures (30 or 31 °C), larvae of *A. colemani* generally cease development [63,64], meaning that the efficacy of this natural enemy will be greatly reduced in summer. Even short periods of high heat can negatively affect populations of *Aphidius* spp. One hour spent at 36 °C decreased *A. avenae* populations by 50% and reduced fitness traits of the survivors [192]. Populations of *A. gossypii* and *M. persicae* can still increase at 30 °C–33 °C [67,73], making aphid biological control difficult at high temperatures.

*Aphidius colemani* can develop at temperatures as low as 10 °C and will still attack aphids at 10–15 °C [63,65]. But, *A. colemani* handling rates decrease linearly with temperature, meaning that 40%–50% fewer aphids are handled at 10–15 than at 20 °C [193]. Given that intrinsic rates of increase of aphids are also much lower at low temperatures [67], successful control of aphids in cooler months is still possible, however, and has been successfully demonstrated by Kim *et al.* [194] in sweet pepper.

Such studies of development times and thresholds do not allow us to predict natural enemy population growth rates at different temperatures, however. This is because a lone metric such as “fast development” can have fitness trade-offs, such as reduction in parasitoid size [66], which also often corresponds to a decrease in female adult longevity and fecundity [66]. Temperature-based population models, e.g., calculations of intrinsic rate of increase (r_m_), are an improvement, since they take into account survival, attack rate, fecundity, and sex ratio along with development time to estimate population growth rate [195]. However, few studies of r_m_ have been conducted for *A. colemani*, and only at a few temperatures (see [17,190,196]). Yet, even these models are limited, as greenhouses do not function at a steady state. Fluctuating daily temperatures and other environmental conditions (see below) are complicating factors in insect development and fecundity (e.g., [67]). Further, insect behavior can be affected by changing environmental conditions, e.g., by shifting activity to parts of the day that are more suitable or increasing patch residence times [197]. Thus, to have any predictive power, more comprehensive models conducted under actual greenhouse conditions are needed. These should include life history studies on the parasitoid wasp, as well as the prey in the presence of the wasp (as in [6]). This should ideally be done on several economically important crop plants, possibly with different architectures (see Section 2.1), grown at different times of year. The results of such studies could potentially be used to predict optimal seasonal usages across plant types.

An unknown environmental effect on *A. colemani* efficacy is the use of “dynamic climate regimes”. This novel method of plant production takes advantage of the adaptability of plants by providing high temperatures during the day, and low temperatures at night (rather than using constant pre-sets) to save energy while still providing optimal long-term average temperatures for plant productivity [198,199]. Recent studies show that this environment management style can reduce heating costs by 10% without negative effects on production [199]. However, this method can also increase *A. gossypii* populations compared to traditional temperature regimes, due to higher short-term mean temperatures favoring aphid development [72]. How *A. colemani* responds in dynamic *versus* constant climate greenhouses remains to be seen, but there are several possible negative effects of this strategy. For example, maximum daytime temperatures may exceed the upper development threshold for *A. colemani*, or reduce the ratio of oviposition activity to other activities, leading to instability in parasitoid-host population dynamics.

Humidity also has important effects on natural enemies in the greenhouse. For example, it can strongly affect *Aphidoletes aphidimyza* (Diptera: Cecidomyiidae) fecundity [200], as well as hatching and predation rates of predatory mites [201,202,203]. Flight and dispersal of *Orius* are highly contingent on temperature and humidity combinations [204,205]. Knowing this, it is surprising that only two papers in the literature have directly addressed humidity effects on an *Aphidius* species. Yan and Chen [206] showed that humidity levels for optimal eclosion and adult longevity for *Aphidius gifuensis* were between 75%–85% RH. At humidity levels above and below this range, eclosion and longevity fell. However, Fink and Volkl [207] did not see differences in foraging abilities (residence times, time allocation, or oviposition success) for *Aphidius rosae* when exposed to low humidity in the field (*ca.* 40% RH). Given the paucity of studies and the disparity between them, this is certainly an important avenue for further investigation. Growers are able to regulate RH to some degree, and currently do so to optimize transpiration or avoid condensation on plants to minimize plant diseases. Control of humidity to optimize *A. colemani* performance may turn out to be an important mediator of aphid biological control. Similarly, effects of “precipitation” in the greenhouse on *A. colemani*, e.g., from misting systems, are currently overlooked. However, simulated light rain reduced foraging of *A. rosae* by >80%, and increase cleaning time by >15% post-rain [207]. Precipitation may also serve to removing searching cues (honeydew) for *A. colemani*.

Recently, new lighting techniques have been a research focus in the greenhouse industry. Offering lower energy costs or improved plant growth, manipulations such as light emitting diodes (LED), photoselective screens (e.g., UV absorbing), and changes in photoperiod may soon become the norm (see review [208]). Given the relative novelty of these techniques, our understanding of how they may affect both pest and natural enemy biology and behavior is far from complete. Both positive and negative effects on various pests and natural enemies have already been noted (see review [209]). For example, several processes that reduce UV-levels are likely improve aphid control, as they have been shown to reduce aphid attraction, dispersal, reproduction and virus transmission [210,211,212]. However, only one study to date has confirmed that reduction of UV light did not interfere with the performance of *A. colemani* [74]. Other lighting techniques and regimes (e.g., changes in day length, light intensity, light quality, *etc.*) also need to be tested to avoid potential disruption of current aphid biological control programs. It is naïve to think that changes in lighting technologies would require no adjustment in pest management, especially since they can alter plant nutritional quality, physical or chemical defenses, and/or volatile emissions or profiles [213,214], which can directly or indirectly affect biological control (see Section 2).

Lastly, wind speed created by cooling fans in greenhouses is also a factor to be considered when using *A. colemani*. Wind speeds of just 2 m/s (4.5 mph) can reduce oviposition and increase resting behavior of the parasitoid *Aphidius rosae* [207], and winds of >0.5 m/s (0.1 mph) can interfere with male mating flights of *Aphidius ervi* and *Aphidius nigripes* [215,216]. A well designed greenhouse has fans that produce wind speeds of 0.9–1.3 m/s (2–3 mph) [217], but many greenhouses operations may be even higher than this in actuality. For the aphid biological control agent *A. aphidimyza*, commercial suppliers often recommend that the fans be turned off for a period of time after release, as wind can interfere with their settling and oviposition behavior [124]. Thus, the potential for improved host seeking and oviposition with a period of no wind should perhaps be investigated for *A. colemani*.

## 6. Conclusions

For over four decades, *A. colemani* has been used for biological control of aphids. This parasitoid is among the most cost effective, and successful biological control agents in greenhouses and is widely used worldwide for controlling *M. persicae* and *A. gossypii*. We identified ecological interactions at each trophic level that can affect *A. colemani* efficacy by affecting either abundance or parasitism rate. Effects present at the 3rd and 4th trophic levels (specifically, the presence of fungal-based insecticides and hyperparasitoids), can clearly have strong, negative effects on *A. colemani* populations. Though these effects can lead to parasitoid population crashes, and potential loss of aphid control, their effects are acute and short term. Proper timing of entomopathogenic fungi sprays and careful management of banker plants could lessen their effects. On the other hand, low quality plants, due to poor nutrition or strong plant defenses, can consistently reduce foraging efficiency and fitness of *A. colemani*. Such effects at the 1st trophic level seem to be the greatest threat to the efficacy of *A. colemani* for aphid control, as they are more chronic, and are more difficult to manage than those posed at other trophic levels. This is because plant traits are often not within a grower’s control (frequently dictated by market demands and limited by current breeding programs). Further, current focus is on optimizing growing conditions for the plant in the most economical way; not necessarily on the wider effects of these production conditions on other trophic levles.

We identified many interactions at each trophic level for which more research is needed. At the first trophic level, more research on how trichome type and density affect *A. colemani* foraging will help predict biological control success in different crops. Further investigation of the net effects of grower practices (e.g., fertilizers, plant growth regulators) on aphid abundance is also necessary, given that such practices can affect plant quality for aphids and parasitoids, and ultimately affect the outcome of biological control programs. Of course, it would be impossible to conduct experiments on each plant species and variety available under different fertilizer regimens. Instead, we need enough research on these interactions to predict general consequences, and make informed recommendations that are applicable to a wide range of crops.

The combined effects of greenhouse temperature, humidity, lighting, and day length on *A. colemani*, and on biological control in general, are also poorly known compared to our knowledge of their effects on plant production [213]. Horticulturalists would never make plant culture recommendations without knowing optimal growing conditions for the plant. Yet, holistic, optimal environmental conditions for effective biological control by a specific beneficial organism are usually not known, and thus not included in recommendations by companies or extension professionals. Basic research on temperatures at which biological control agents die or become inactive is often available, and, in some cases, even the optimum range for parasitism rate is known. However, this information has limited usefulness in a vacuum. Future research needs to more clearly recognize that temperature and other environmental factors also change pest population growth rates in concert with parasitoid life history and behavior. Again, we need to have enough research to predict the net effect of increasing or decreasing temperature (or other conditions) on pest abundance in the presence of a natural enemy, not just on isolated factors such as parasitism rates. Further, research surrounding abiotic factors on aphids and their natural enemies should include both research on the greater greenhouse environment, as well as how this relates to the microclimate within the plant canopy where arthropods organisms generally function [213].

Genetic analysis of commercial and wild *A. colemani* is an important avenue for research. This will be important to reduce inbreeding, and to develop “strains” that are easy to rear but without negative trade-offs such as small size. Commercial insectaries may want to consider capitalizing on traits of wild populations, as potentially useful variations in life history characteristics have been identified in parasitoid populations outside of greenhouses (see [191]). Further, confirming the identity and characteristics of commercial populations of *A. colemani* should be an industry priority. Should *A. colemani* shipments prove to be a mix of cryptic species, then any research predictions based on the biology or behavior of a single population would be hopelessly muddled.

Since the 1970s researchers and growers have learned much about *A. colemani*. Our review demonstrates the abundance and complexity of interactions that could affect *A. colemani* efficacy. Many of these interactions are driven by plant selection, grower inputs, and the abiotic environment which directly and indirectly affect plant quality for pests and parasitoids. The research needed to advance our understanding and recommendations will require an interdisciplinary approach rather than entomologists working in isolation. For example, working with plant breeders could result in plant varieties that strike a balance between consumer-valued traits, pest resistance traits, and traits that are favorable to biological control. Likewise, working with horticulturalists could help guide plant growth regulator and fertilizer recommendations toward those that reduce positive effects on pests or negative effects on parasitoids. Collaboration between researchers, extension personnel, the pest control industry, and growers will be essential to advance *A. colemani* efficacy and support the growing use of biological control in greenhouses.

## References

[1] Messing R., Rabasse J.M. (1995). Oviposition behaviour of the polyphagous aphid parasitoid *Aphidius colemani* Viereck (Hymenoptera: Aphidiidae). Agric. Ecosyst. Environ..

[2] Starý P. (1975). *Aphidius colemani* Viereck: its taxonomy, distribution and host range (Hymenoptera, Aphidiidae). Acta Entomol. Bohemoslov..

[3] Benelli G., Messing R.H., Wright M.G., Giunti G., Kavallieratos N.G., Canale A. (2014). Cues triggering mating and host-seeking behavior in the aphid parasitoid *Aphidius colemani* (Hymenoptera: Braconidae: Aphidiinae): Implications for biological control. J. Econ. Entomol..

[4] Van Steenis M.J., El-Khawass K.A.M.H. (1995). Behaviour of *Aphidius colemani* searching for aphis gossypii: Functional response and reaction to previously searched aphid colonies. Biocontrol Sci. Technol..

[5] Bilu E., Hopper K.R., Coll M. (2006). Host choice by *Aphidius colemani*: Effects of plants, plant-aphid combinations and the presence of intra-guild predators. Ecol. Entomol..

[6] Vásquez G.M., Orr D.B., Baker J.R. (2006). Efficacy assessment of *Aphidius colemani* (Hymenoptera: Braconidae) for suppression of *Aphis gossypii* (Homoptera: Aphididae) in greenhouse-grown chrysanthemum. J. Econ. Entomol..

[7] Boivin G., Hance T., Brodeur J. (2012). Aphid parasitoids in biological control. Can. J. Plant Sci..

[8] Van Schelt J., Hoogerbrugge H., Becker N., Messelink G.J., Blockmans K. (2011). Comparing *Aphidius colemani* and *Aphidius matricariae* on *Myzus persicae* ssp. nicotianae in sweet pepper. IOBC/WPRS Bull..

[9] Chau A., Heinz K., Heinz K.M., van Driesche R.G., Parella M.P. (2001). Biological control of aphids on ornamental crops. Biocontrol in Protected Culture.

[10] Yano E. (2006). Ecological considerations for biological control of aphids in protected culture. Popul. Ecol..

[11] Van Lenteren J. (1988). Biological and integrated pest control in greenhouses. Annu. Rev. Entomol..

[12] Heinz K.M. (1998). Dispersal and dispersion of aphids (Homoptera: Aphididae) and selected natural enemies in spatially subdivided greenhouse environments. Environ. Entomol..

[13] Bennison J.A., Corless S.P. (1992). Biological control of aphids on cucumbers: further development of open rearing units or “banker plants” to aid the establishment of aphid natural enemies. IOBC/WPRS Bull..

[14] Van Steenis M.J. (1995). Evaluation of four aphidiine parasitoids for biological control of Aphis gossypii. Entomol. Exp. Appl. Appl..

[15] Van Driesche R.G., Lyon S., Sanderson J.P.S., Bennett K.C., Stanek E.J.I., Zhang R. (2008). Greenhouse Trials of *Aphidius colemani* (Hymenoptera: Braconidae) banker plants for control of aphids (Hemiptera: Aphididae) in greenhouse spring floral crops. Florida Entomol..

[16] Frank S.D. (2010). Biological control of arthropod pests using banker plant systems: Past progress and future directions. Biol. Control.

[17] Van Steenis M.J. (1993). Intrinsic rate of increase of *Aphidius colemani* Vier. (Hym., Braconidae), a parasitoid of *Aphis gossypii* Glov. (Hom., Aphididae), at different temperatures. J. Appl. Entomol..

[18] Burgio G., Ferrari R., Giorgio N. Biological and integrated control of *Aphis gossypii* Glove (Hom., Aphidide) in protected cucumber and melon. http://www.bulletinofinsectology.org/pdfarticles/vol51-1997-171-178burgio.pdf.

[19] Jacobson R.J., Croft P. (1998). Strategies for the control of *Aphis gossypii* Glover (Hom.: Aphididae) with *Aphidius colemani* Viereck (Hym.: Braconidae) in protected cucumbers. Biocontrol Sci. Technol..

[20] Nagasaka K., Takahasi N., Okabayashi T. (2010). Impact of secondary parasitism on *Aphidius colemani* in the banker plant system on aphid control in commercial greenhouses in Kochi, Japan. Appl. Entomol. Zool..

[21] Acheampong S., Gillespie D.R., Quiring D. (2012). Survey of parasitoids and hyperparasitoids (Hymenoptera) of the green peach aphid, *Myzus persicae* and the foxglove aphid, *Aulacorthum solani* (Hemiptera: Aphididae) in British Columbia. J. Entomol. Soc. Br. Columbia.

[22] Jandricic S.E. (2015). Personal Observation.

[23] Aalbers J. (2015). Personal communication.

[24] Holt R. (1977). Predation, apparent competition, and the structure of prey communities. Theor. Popul. Biol..

[25] Desneux N., Ramirez-Romero R. (2009). Plant characteristics mediated by growing conditions can impact parasitoid’s ability to attack host aphids in winter canola. J. Pest Sci..

[26] Prado S.G., Frank S.D. (2013). Compact plants reduce biological control of *Myzus persicae* by *Aphidius colemani*. Biol. Control.

[27] Prado S.G., Frank S.D. (2013). Tritrophic effects of plant growth regulators in an aphid-parasitoid system. Biol. Control.

[28] Grasswitz T.R. (1998). Effect of adult experience on the host-location behavior of the aphid parasitoid *Aphidius colemani* Viereck (Hymenoptera: Aphidiidae). Biol. Control.

[29] Storeck A., Poppy G.M., van Emden H.F., Powell W. (2000). The role of plant chemical cues in determining host preference in the generalist aphid parasitoid *Aphidius colemani*. Entomol. Exp. Appl..

[30] Douloumpaka S., van Emden H.F. (2003). A maternal influence on the conditioning to plant cues of *Aphidius colemani* Viereck, parasitizing the aphid *Myzus persicae* Sulzer. Physiol. Entomol..

[31] Lo Pinto M., Wajnberg E., Colazza S., Curty C., Fauvergue X. (2004). Olfactory response of two aphid parasitoids, *Lysiphlebus testaceipes* and *Aphidius colemani*, to aphid-infested plants from a distance. Entomol. Exp. Appl..

[32] Kalule T., Wright D.J. (2004). The influence of cultivar and cultivar-aphid odours on the olfactory response of the parasitoid *Aphidius colemani*. J. Appl. Entomol..

[33] Charles-Tollerup J.J. Resource Provisioning as a Habitat Manipulation Tactic to Enhance the Aphid Parasitoid, *Aphidius colemani* Viereck (Hymenoptera: Braconidae: Aphidiinae),and the Plant-Mediated Effects of a Systemic Insecticide, Imidacloprid. https://escholarship.org/uc/item/97w046gw#page-1.

[34] Aqueel M.A., Raza A.M., Balal R.M., Shahid M.A., Mustafa I., Javaid M.M., Leather S.R. (2014). Tritrophic interactions between parasitoids and cereal aphids are mediated by nitrogen fertilizer. Insect Sci..

[35] Jandricic S.E., Dale A.G., Bader A., Frank S.D. (2014). The effect of banker plant species on the fitness of *Aphidius colemani* Viereck and its aphid host (*Rhopalosiphum padi* L.). Biol. Control.

[36] Schädler M., Brandl R., Kempel A. (2010). Host plant genotype determines bottom-up effects in an aphid-parasitoid-predator system. Entomol. Exp. Appl..

[37] Ode P.J., Hopper K.R., Coll M. (2005). Oviposition *vs.* offspring fitness in *Aphidius colemani* parasitizing different aphid species. Entomol. Exp. Appl..

[38] Prado S.G., Frank S.D. (2014). Optimal foraging by an aphid parasitoid affects the outcome of apparent competition. Ecol. Entomol..

[39] Vorburger C., Gehrer L., Rodriguez P. (2009). A strain of the bacterial symbiont Regiella insecticola protects aphids against parasitoids.

[40] Gillespie D.R., Quiring D.J.M., Foottit R.G., Foster S.P., Acheampong S. (2009). Implications of phenotypic variation of *Myzus persicae* (Hemiptera: Aphididae) for biological control on greenhouse pepper plants. J. Appl. Entomol..

[41] Perdikis D.C., Lykouressis D.P., Garantonakis N.G., Iatrou S.A. (2004). Instar preference and parasitization of *Aphis gossypii* and *Myzus persicae* (Hemiptera: Aphididae) by the parasitoid *Aphidius colemani* (Hymenoptera: Aphidiidae). Eur. J. Entomol..

[42] Martinou A.F., Wright D.J. (2007). Host instar and host plant effects on *Aphidius colemani*. J. Appl. Entomol..

[43] Lin L.A., Ives A.R. (2003). The effect of parasitoid host-size preference on host population growth rates: An example of *Aphidius colemani* and *Aphis glycines*. Ecol. Entomol..

[44] Wu G.-M., Barrette M., Boivin G., Brodeur J., Giraldeau L., Hance T., Wu A.G. (2011). Temperature influences the handling efficiency of an aphid parasitoid through body size-mediated effects. Environ. Entomol..

[45] Lykouressis D., Garantonakis N., Perdikis D., Fantinou A., Mauromoustakos A. (2009). Effect of female size on host selection by a koinobiont insect parasitoid (Hymenoptera: Braconidae: Aphidiinae). Eur. J. Entomol..

[46] Stadler B., Volkl W. (1991). Foraging patterns of two aphid parasitoids, *Lysiphlebus testaceipes* and *Aphidius colemani* on banana. Entomol. Exp. Appl..

[47] Wäckers F.L., van Rijn P.C.J., Heimpel G.E. (2008). Honeydew as a food source for natural enemies: Making the best of a bad meal?. Biol. Control.

[48] Sampaio M.V., Bueno V.H.P., Soglia M., da C., de M., de Conti B.F., Rodrigues S.M.M. (2006). Larval competition between *Aphidius colemani* and *Lysiphlebus testaceipes* after multiparasitism of the host *Aphis gossypii*. Bull. Insectology.

[49] Brodeur J., Rosenheim J.A. (2000). Intraguild interactions in aphid parasitoids. Entomol. Exp. Appl..

[50] Bilu E., Coll M. (2007). The importance of intraguild interactions to the combined effect of a parasitoid and a predator on aphid population suppression. BioControl.

[51] Messelink G.J., Bloemhard C.M.J., Sabelis M.W., Janssen A. (2012). Biological control of aphids in the presence of thrips and their enemies. BioControl.

[52] Shipp J.L., Zhang Y., Hunt D.W.A., Ferguson G. (2003). Influence of humidity and greenhouse Microclimate on the efficacy of *Beauveria bassiana* (Balsamo) for control of greenhouse arthropod pests. Environ. Entomol..

[53] Ludwig S.W., Oetting R.D. (2001). Susceptibility of natural enemies to infection by *Beauveria bassiana* and impact of insecticides on Ipheseius degenerans Acari: Phytoseiidae. J. Agric. Urban Entomol..

[54] Kim J.J., Kim K.C., Roberts D.W. (2005). Impact of the entomopathogenic fungus *Verticillium lecanii* on development of an aphid parasitoid, *Aphidius colemani*. J. Invertebr. Pathol..

[55] Baverstock J., Alderson P.G., Pell J. (2005). Influence of the aphid pathogen *Pandora neoaphidis* on the foraging behaviour of the aphid parasitoid *Aphidius ervi*. Ecol. Entomol..

[56] Grasswitz T.R., Reese B.D. (1998). Biology and host selection behaviour of the aphid hyperparasitoid *Alloxysta victrix* in association with the primary parasitoid *Aphidius colemani* and the host aphid *Myzus persicae*. BioControl.

[57] Bloemhard C.M.J., van der Wielen M., Messelink G.J. (2014). Seasonal abundance of aphid hyperparasitoids in organic greenhouse crops in The Netherlands. IOBC/WPRS Bull..

[58] Dinter A., Wiles J.A. (2000). Safety of the new DuPont insecticide indoxicarb to beneficial arthropods: An overview. IOBC/WPRS Bull..

[59] Bostanian N., Akalach M., Chiasson H. (2005). Effects of a Chenopodium-based botanical insecticide/acaracide on *Orius insidiosus* (Hemiptera: Anthocoridae) and *Aphidus colemani* (Hymenoptera: Braconidae). Pest Manag. Sci..

[60] Takahashi Y., Kojimoto T., Nagaoka H., Takagi Y., Oikawa M. (2005). Evaluating the side effects of chlorothalonil (TPN) and spinosad on the parasitic wasp (*Aphidius colemani*). J. Pestic. Sci..

[61] Tremblay É., Bélanger A., Brosseau M., Boivin G. (2008). Toxicity and sublethal effects of an insecticidal soap on *Aphidius colemani* (Hymenoptera: Braconidae). Pest Manag. Sci..

[62] Stara J., Ourednickova J., Kocourek F. (2010). Laboratory evaluation of the side effects of insecticides on *Aphidius colemani* (Hymenoptera: Aphidiidae), *Aphidoletes aphidimyza* (Diptera: Cecidomyiidae), and *Neoseiulus cucumeris* (Acari: Phytoseidae). J. Pest Sci..

[63] Goh H.G., Kim J.H., Han M.W. (2001). Application of *Aphidius colemani* viereck for control of the aphid in greenhouse. J. Asia. Pac. Entomol..

[64] Bueno V.H.P., Sampaio M.V., van Lenteren J.C., De Conti B.F., Silva R.J., Rodrigues S.M.M., Carnevale A.B., Castañé C., Sanchez J.A. (2006). Evaluation of two aphid parasitoids as candidates for biocontrol of aphid pests in protected cultivation in Brazil. Integrated Control in Protected Crops, Mediterranean Climate.

[65] Zamani A.A., Talebi A., Fathipour Y., Baniameri V. (2007). Effect of temperature on life history of *Aphidius colemani* and *Aphidius matricariae* (Hymenoptera: Braconidae), two parasitoids of *Aphis gossypii* and *Myzus persicae* (Homoptera: Aphididae). Environ. Entomol..

[66] Colinet H., Boivin G., Hance T. (2007). Manipulation of parasitoid size using the temperature-size rule: Fitness consequences. Oecologia.

[67] Davis J.A., Radcliffe E.B., Ragsdale D.W. (2006). Effects of high and fluctuating temperatures on *Myzus persicae* (Hemiptera: Aphididae). Environ. Entomol..

[68] Shipp J.L., Wang K., Ferguson G. (2000). Residual toxicity of avermectin b1 and pyridaben to eight commercially produced beneficial arthropod species used for control of greenhouse pests. Biol. Control.

[69] Smith T. Biological control: Pesticide compatibility, testing quality and storage. https://ag.umass.edu/fact-sheets/biological-control-pesticide-compatibility-testing-quality-storage.

[70] Langhof M., Gathmann A., Poehling H.M., Meyhöfer R. (2003). Impact of insecticide drift on aphids and their parasitoids: Residual toxicity, persistence and recolonisation. Agric. Ecosyst. Environ..

[71] Bostanian N.J., Akalach M. (2004). The contact toxicity of indoxacarb and five other insecticides to *Orius insidiosus* (Hemiptera: Anthocoridae) and *Aphidius colemani* (Hymenoptera: Braconidae), beneficials used in the greenhouse industry. Pest Manag. Sci..

[72] Jakobsen L., Brogaard M., Körner O., Enkegaard A., Aaslyng J.M. (2005). The influence of a dynamic climate on pests, diseases and beneficial organisms: Recent research. IOBC WPRS Bull..

[73] Satar S., Kersting U., Uygun N. (2005). Effect of temperature on development and fecundity of *Aphis gossypii* Glover (Homoptera: Aphididae) on cucumber. J. Pest Sci..

[74] Chiel E., Messika Y., Steinberg S., Antignus Y. (2006). The effect of UV-absorbing plastic sheet on the attraction and host location ability of three parasitoids: *Aphidius colemani*, *Diglyphus isaea* and *Eretmocerus mundus*. Biocontrol.

[75] Price P.W., Bouton C.E., Gross P., McPheron B.A., Thompson J.N., Weis A.E. (1980). Interactions among three trophic levels: Influence of plants on interactions between insect herbivores and natural enemies. Annu. Rev. Ecol. Syst..

[76] Kareiva P., Sahakian R. (1990). Tritrophic effects of a simple architectural mutation in pea plants. Nature.

[77] Chang G.C., Neufeld J., Durr D., Duetting P.S., Eigenbrode S.D. (2004). Waxy bloom in peas influences the performance and behavior of *Aphidius ervi*, a parasitoid of the pea aphid. Entomol. Exp. Appl..

[78] Duetting P.S., Ding H., Neufeld J., Eigenbrode S.D. (2003). Plant waxy bloom on peas affects infection of pea aphids by *Pandora neoaphidis*. J. Invertebr. Pathol..

[79] Eigenbrode S.D. (2004). The effects of plant epicuticular waxy blooms on attachment and effectiveness of predatory insects. Arthropod Struct. Dev..

[80] Gamarra D.C., Bueno V.H.P., Auad A.M. (1997). Efecto de los tricomas glandulares de Solanum berthaultii en el parasitismo de *Aphidius colemani* (Hymenoptera: Aphidiidae) sobre *Myzus persicae* (Homoptera: Aphididae). Vedalia.

[81] Cloyd R.A., Sadof C.S. (2000). (Hymenoptera: Encyrtidae), a Parasitoid of the *Citrus Mealybug* (Homoptera: Pseudococcidae). Environ. Entomol..

[82] Gingras D. (2003). Effect of plant structure on host finding capacity of lepidopterous pests of crucifers by two Trichogramma parasitoids. Biol. Control.

[83] Andow D.A., Prokrym D.R. (1990). Plant structural complexity and host-finding by a parasitoid. Oecologia.

[84] Gontijo L.M., Margolies D.C., Nechols J.R., Cloyd R.A. (2010). Plant architecture, prey distribution and predator release strategy interact to affect foraging efficiency of the predatory mite *Phytoseiulus persimilis* (Acari: Phytoseiidae) on cucumber. Biol. Control.

[85] Gingras D., Dutilleul P., Boivin G. (2002). Modeling the impact of plant structure on host-finding behavior of parasitoids. Oecologia.

[86] Vet L.E.M. (2001). Parasitoid searching efficiency links behaviour to population processes. Appl. Entomol. Zool..

[87] Van Emden H.F., Eletherianos I., Rose J., Douloumpaka S., Pettersson J. (2002). Aphid parasitoids detect that an alien plant was present nearby during their development. Physiol. Entomol..

[88] Tylianakis J.M., Didham R.K., Wratten S.D. (2004). Improved fitness of aphid parasitoids receiving resource subsidies. Ecology.

[89] Araj S.A., Wratten S.D., Lister A.J., Buckley H.L. (2006). Floral nectar affects longevity of the aphid parasitoid *Aphidius ervi* and its hyperparasitoid *Dedrocerys aphidium*. New Zeal. Plant Prot..

[90] Berndt L.A., Wratten S.D., Scarratt S.L. (2006). The influence of floral resource subsidies on parasitism rates of leafrollers (Lepidoptera: Tortricidae) in New Zealand vineyards. Biol. Control.

[91] Helenius J. (1990). Effect of epigeal predators on infestation by the aphid *Rhopalosiphum padi* and on grain yield of oats in monocrops and mixed intercrops. Entomol. Exp. Appl..

[92] Baggen L.R., Gurr G.M., Meats A. (1999). Flowers in tri-trophic systems: Mechanisms allowing selective exploitation by insect natural enemies for conservation biological control. Entomol. Exp. Appl..

[93] Baggen L.R., Gurr G.M. (1998). The influence of food on *Copidosoma koehleri* (Hymenoptera: Encyrtidae), and the use of flowering plants as a habitat management tool to enhance biological control of potato moth, *Phthorimaea operculella* (Lepidoptera: Gelechiidae). Biol. Control.

[94] Romeis J., Wäckers F.L. (2000). Feeding responses by female Pieris brassicae butterflies to carbohydrates and amino acids. Physiol. Entomol..

[95] Awmack C.S., Leather S.R. (2002). Host plant quality and fecundity in herbivorous insects. Annu. Rev. Entomol..

[96] Eigenbrode S.D., Trumble J.T. (1994). Host plant resistance to insects in integrated pest management in vegetable crops. J. Agric. Entomol..

[97] Ode P.J. (2006). Plant chemistry and natural enemy fitness: Effects on herbivore and natural enemy interactions. Annu. Rev. Entomol..

[98] Bottrell D.G., Barbosa P., Gould F. (1998). Manipulating natural enemies by plant variety selection and modification: A realistic strategy?. Annu. Rev. Entomol..

[99] Kauffman W.C., Flanders R.V., Edwards C.R. (1985). Effects of variably resistant soybean and lima bean cultivars on *Pediobius foveolatus*, a parasitoid of the Mexican bean beetle, *Epilachna varivestis*. Environ. Entomol..

[100] Ode P.J., Berenbaum M.R., Zangerl A.R., Hardy I.C.W. (2004). Host plant, host plant chemistry and the polyembryonic parasitoid *Copidosoma sosares*: Indirect effects in a tritrophic interaction. Oikos.

[101] Chen Y., Olson D.M., Ruberson J.R. (2010). Effects of nitrogen fertilization on tritrophic interactions. Arthropod. Plant. Interact..

[102] Throop H.L., Lerdau M.T. (2004). Effects of nitrogen deposition on insect herbivory: Implications for community and ecosystem processes. Ecosystems.

[103] Krauss J., Härri S.A., Bush L., Husi R., Bigler L., Power S.A., Müller C.B. (2007). Effects of fertilizer, fungal endophytes and plant cultivar on the performance of insect herbivores and their natural enemies. Funct. Ecol..

[104] Zarghami S., Allahyari H., Bagheri M.R., Saboori A. (2010). Effect of nitrogen fertilization on life table parameters and population growth of *Brevicoryne brassicae*. Bull. Insectology.

[105] Couture J.J., Servi J.S., Lindroth R.L. (2010). Increased nitrogen availability influences predator-prey interactions by altering host-plant quality. Chemoecology.

[106] Pope T.W., Girling R.D., Staley J.T., Trigodet B., Wright D.J., Leather S.R., van Emden H.F., Poppy G.M. (2012). Effects of organic and conventional fertilizer treatments on host selection by the aphid parasitoid *Diaeretiella rapae*. J. Appl. Entomol..

[107] Staley J.T., Girling R.D., Stewart-Jones A., Poppy G.M., Leather S.R., Wright D.J. (2011). Organic and conventional fertilizer effects on a tritrophic interaction: Parasitism, performance and preference of *Cotesia vestalis*. J. Appl. Entomol..

[108] Staley J.T., Stewart-Jones A., Poppy G.M., Leather S.R., Wright D.J. (2009). Fertilizer affects the behaviour and performance of *Plutella xylostella* on brassicas. Agric. For. Entomol..

[109] Kos M., Houshyani B., Achhami B.B., Wietsma R., Gols R., Weldegergis B.T., Kabouw P., Bouwmeester H.J., Vet L.E.M., Dicke M., van Loon J.J. (2012). Herbivore-mediated effects of glucosinolates on different natural enemies of a specialist aphid. J. Chem. Ecol..

[110] Chow A., Chau A., Heinz K.M. (2009). Reducing fertilization for cut roses: Effect on crop productivity and twospotted spider mite abundance, distribution, and management. J. Econ. Entomol..

[111] Chow A., Chau A., Heinz K.M. (2012). Reducing fertilization: A management tactic against western flower thrips on roses. J. Appl. Entomol..

[112] Härri S.A., Krauss J., Müller C.B. (2008). Trophic cascades initiated by fungal plant endosymbionts impair reproductive performance of parasitoids in the second generation. Oecologia.

[113] Herman M.A.B., Nault B.A., Smart C.D. (2008). Effects of plant growth-promoting rhizobacteria on bell pepper production and green peach aphid infestations in New York. Crop Prot..

[114] Boutard-Hunt C., Smart C.D., Thaler J., Nault B.A. (2009). Impact of plant growth-promoting rhizobacteria and natural enemies on *Myzus persicae* (Hemiptera: Aphididae) infestations in pepper. J. Econ. Entomol..

[115] Pineda A., Soler R., Weldegergis B.T., Shimwela M.M., Van Loon J.J., Dicke M. (2013). Non-pathogenic rhizobacteria interfere with the attraction of parasitoids to aphid-induced plant volatiles via jasmonic acid signalling. Plant. Cell Environ..

[116] Zytynska S.E., Fleming S., Tetard-Jones C., Kertesz M.A., Preziosi R. (2010). Community genetic interactions mediate indirect ecological effects between a parasitoid wasp and rhizobacteria. Ecology.

[117] Schardl C.L., Leuchtmann A., Spiering M.J. (2004). Symbioses of grasses with seedborne fungal endophytes. Annu. Rev. Plant Biol..

[118] Härri S.A., Krauss J., Müller C.B. (2009). Extended larval development time for aphid parasitoids in the presence of plant endosymbionts. Ecol. Entomol..

[119] Strand M.R., Obrycki J.J. (1996). Host specificity of insect parasitoids and predators. Bioscience.

[120] Rehman A., Powell W. (2010). Host selection behaviour of aphid parasitoids (Aphidiidae: Hymenoptera). J. Plant Breed. Crop Sci..

[121] Sampaio M.V., Bueno V.H.P., De Conti B.F. (2008). The effect of the quality and size of host aphid species on the biological characteristics of *Aphidius colemani* (Hymenoptera: Braconidae: Aphidiinae). Eur. J. Entomol..

[122] Visser M.E. (1994). The importance of being large: The relationship between size and fitness in females of the parasitoid *Aphaereta minuta* (Hymenoptera: Braconidae). J. Anim. Ecol..

[123] Eijs I.E.M., van Alphen J.J.M. (1999). Life history correlations: Why are hymenopteran parasitoids an exception?. Ecol. Lett..

[124] Jandricic S.E. Investigations of the Biology of the Pest Aphid Aulacorthum Solani (Kaltenbach) (Hemiptera: Aphididae) And Of Biological Control Agents For Control Of Multi-Species Aphid Outbreaks In Greenhouse Floriculture Crops. https://dspace.library.cornell.edu/bitstream/1813/34184/1/sej48.pdf.

[125] Oliver K.M., Russell J., Moran N., Hunter M.S. (2003). Facultative bacterial symbionts in aphids confer resistance to parasitic wasps. Proc. Natl. Acad. Sci. USA.

[126] Oliver K.M., Moran N., Hunter M.S. (2005). Variation in resistance to parasitism in aphids is due to symbionts not host genotype. Proc. Natl. Acad. Sci. USA.

[127] Kovacs J., Gaul C., Wolf S., Voisin D., Gerardo N. The Effects of Endosymbionts Across Food Webs: How Aphid Endosymbionts Affect the Survival of the Predatory Invasive Lady Beetle Harmonia axyridis. http://www.xcdsystem.com/evolution2014/abstract/abstractforms/screen_view_abstract_public.cfm?ID=20067.

[128] Costopoulos K., Kovacs J.L., Kamins A., Gerardo N.M. (2014). Aphid facultative symbionts reduce survival of the predatory lady beetle *Hippodamia convergens*. BMC Ecol..

[129] Koga R., Tsuchida T., Sakurai M., Fukatsu T. (2007). Selective elimination of aphid endosymbionts: Effects of antibiotic dose and host genotype, and fitness consequences. FEMS Microbiol. Ecol..

[130] Bergeson E., Messina F.J. (1998). Effect of a co-occurring aphid on the susceptibility of the Russian wheat aphid to lacewing predators. Entomol. Exp. Appl..

[131] Sampaio M.V., Bueno V.H.P., Perez-Maluf R. (2001). Parasitismo de *Aphidius colemani* Viereck (Hymenoptera: Aphidiidae) em Diferentes Densidades de *Myzus persicae* (Sulzer) (Hemiptera: Aphididae). Neotrop. Entomol..

[132] Murphy G. (2015). Personal Communication.

[133] Benelli G., Kavallieratos N.G., Donati E., Mencattelli M., Bonsignori G., Stefanini C., Canale A., Messing R.H. (2014). May the wild male loose? Male wing fanning performances and mating success in wild and mass-reared strains of the aphid parasitoid *Aphidius colemani* Viereck (Hymenoptera: Braconidae: Aphidiinae). BioControl.

[134] Jandricic S.E., Wraight S.P., Gillespie D.R., Sanderson J.P. (2013). Oviposition behavior of the biological control agent *Aphidoletes aphidimyza* (Diptera: Cecidomyiidae) in environments with multiple pest aphid species (Hemiptera: Aphididae). Biol. Control.

[135] Meisner M., Harmon J.P., Ives A.R. (2007). Presence of an unsuitable host diminishes the competitive superiority of an insect parasitoid: A distraction effect. Popul. Ecol..

[136] Michaud J.P., Mackauer M. (1994). The use of visual cues in host evaluation by aphidiid wasps—I. Comparison between three Aphidius parasitoids of the pea aphid. Entomol. Exp. Appl..

[137] Foster S.P., Tomiczek M., Thompson R., Denholm I., Poppy G., Kraaijeveld A.R., Powell W. (2007). Behavioural side-effects of insecticide resistance in aphids increase their vulnerability to parasitoid attack. Anim. Behav..

[138] Foster S.P., Kift N.B., Baverstock J., Sime S., Reynolds K., Jones J.E., Thompson R., Tatchell G.M. (2003). Association of MACE-based insecticide resistance in *Myzus persicae* with reproductive rate, response to alarm pheromone and vulnerability to attack by *Aphidius colemani*. Pest Manag. Sci..

[139] Karagounis C., Kourdoumbalos K., Margaritopoulos J.T., Nanos G.D., Tsitsipis J. (2006). Organic farming-compatible insecticides against the aphid *Myzus persicae* (Sulzer) in peach orchards. J. Appl. Entomol..

[140] Colinet H., Salin C., Boivin G., Hance T. (2005). Host age and fitness-related traits in a koinobiont aphid parasitoid. Ecol. Entomol..

[141] Le Ralec A., Anselme C., Outreman Y., Poirié M., van Baaren J., le Lann C., van Alphen J.J.-M. (2010). Evolutionary ecology of the interactions between aphids and their parasitoids. C. R. Biol..

[142] Gerling D., Roitberg B.D., Mackauer M. (1990). Instar-specific defense of the pea aphid, *Acyrthosiphon pisum*: Influence on oviposition success of the parasite *Aphelinus asychis* (Hymenoptera: Aphelmidae). J. Insect Behav..

[143] Paul G. (1993). Insect behavioral and morphological defenses against parasitoids. Annu. Rev. Entomol..

[144] Henry L.M., Ma B.O., Roitberg B.D. (2009). Size-mediated adaptive foraging: A host-selection strategy for insect parasitoids. Oecologia.

[145] Michaud J.P., Mackauer M. (1995). The use of visual cues in host evaluation by aphidiid wasps: II. Comparison between *Ephedrus californicus*, *Monoctonus paulensis*, and *Praon pequodorum*. Entomol. Exp. Appl..

[146] Henry L.M., Roitberg B.D., Gillespie D.R. (2006). Covariance of phenotypically plastic traits induces an adaptive shift in host selection behaviour. Proc. Biol. Sci..

[147] Byeon Y.W., Tuda M., Kim J.H., Choi M.Y. (2011). Functional responses of aphid parasitoids, *Aphidius colemani* (Hymenoptera: Braconidae) and *Aphelinus asychis* (Hymenoptera: Aphelinidae). Biocontrol Sci. Technol..

[148] Bouchard Y., Cloutier C. (1984). Honeydew as a source of host-searching kairomones for the aphid parasitoid *Aphidius nigripes* (Hymenoptera: Aphidiidae). Can. J. Zool..

[149] Chow F.J., Mackauer M. (1984). Inter- and intraspecific larval competion in *Aphidius smithi* and *Praon pequodorum* (Hymenoptera: Aphididae). Can. Entomol..

[150] McBrien H., Mckauer M. (1990). Heterospecific larval competition and host discrimination in two species of aphid parasitoids: *Aphidius ervi* and *Aphidius smithi*. Entomol. Exp. Appl..

[151] Tomanović T.Z., Petrović A., Mitrović M., Kavallieratos N.G., Starý P., Rakhshani E., Rakhshanipour M., Popović A., Shukshu A.H., Ivanović A. (2014). Molecular and morphological variability within the *Aphidius colemani* group with redescription of *Aphidius platensis* Brethes (Hymenoptera: Braconidae: Aphidiinae). Bull. Entomol. Res..

[152] Frewin A.J., Murphy C.S.-D.G., Hanner R. (2014). DNA barcoding of commercial biological control agents: A quality management framework. IOBC WPRS Bull..

[153] Meyhofer R., Klug T. (2002). Intraguild predation on the aphid parasitoid *Lysiphlebus fabarum* (Marshall) (Hymenoptera : Aphidiidae): Mortality risks and behavioral decisions made under the threats of predation. Biol. Control.

[154] Snyder W.E., Ives A.R. (2003). Interactions between specialist and generalist natural enemies: Parasitoids, predators, and pea aphid biocontrol. Ecology.

[155] Almohamad R., Verheggen F.J., Francis F., Hance T., Haubruge E. (2008). Discrimination of parasitized aphids by a hoverfly predator: effects on larval performance, foraging, and oviposition behavior. Entomol. Exp. Appl..

[156] Martinou A.F., Raymond B., Milonas P.G., Wright D.J. (2010). Impact of intraguild predation on parasitoid foraging behaviour. Ecol. Entomol..

[157] Ferguson K.I., Stiling P. (1996). Non-additive effects of multiple natural enemies on aphid populations. Oecologia.

[158] Snyder W.E., Ives A.R. (2001). Generalist predators disrupt biological control by a specialist parasitoid. Ecology.

[159] Chacón J.M., Landis D., Heimpel G.E. (2008). Potential for biotic interference of a classical biological control agent of the soybean aphid. Biol. Control.

[160] Colfer R.G., Rosenheim J. (2001). Predation on immature parasitoids and its impact on aphid suppression. Oecologia.

[161] Costamagna A.C., Landis D.A., Difonzo C.D. (2007). Suppression of soybean aphid by generalist predators results in a trophic cascade in soybeans. Ecol. Appl..

[162] Straub C.S., Snyder W.E. (2008). Increasing enemy biodiversity strengthens herbivore suppression on two plant species. Ecology.

[163] Weisser W.W., Soares A.O., Ventura M.A., Garcia V., Hemptinne J.-L. (2003). Additive effects of pea aphid natural enemies despite intraguild predation. Proceedings of the 8th International Sympoisum on Ecolgoy of Aphidophaga: Biology, Ecology and Behavior of Aphidophagous Insects.

[164] Snyder W.E., Ballard S.N., Yang S., Clevenger G.M., Miller T.D., Ahn J.J., Hatten T.D., Berryman A. (2004). Complementary biocontrol of aphids by the ladybird beetle *Harmonia axyridis* and the parasitoid *Aphelinus asychis* on greenhouse roses. Biol. Control.

[165] Pilkington L.J., Messelink G., van Lenteren J.C., le Mottee K. (2010). “Protected Biological Control”—Biological pest management in the greenhouse industry. Biol. Control.

[166] Li T., Liu Y., Zhang Y. (2007). Impacts of *Beauveria bassiana* on life parameters and control efficiency of *Aphidius gifuensis*. Mycosystema.

[167] Fuentes-Contreras E., Pell J.K., Niemeyer H.M. (1998). Influence of plant resistance at the third trophic level: Interactions between parasitoids and entomopathogenic fungi of cereal aphids. Oecologia.

[168] Powell W., Wilding N., Brobyn P.J., Clark S.J. (1986). Interference between parasitoids (Hym.: Aphidiidae) and fungi (Entomophthorales) attacking cereal aphids. Entomophaga.

[169] Jandricic S.E., Filotas M., Sanderson J.P., Wraight S.P. (2014). Pathogenicity of conidia-based preparations of entomopathogenic fungi against the greenhouse pest aphids *Myzus persicae*, *Aphis gossypii*, and *Aulacorthum solani* (Hemiptera: Aphididae). J. Invertebr. Pathol..

[170] Kanagaratnam P., Hall R.A., Burges H.D. (1982). Control of glasshouse whitefly, *Trialeurodes vaporariorum*, by an “aphid” strain of the fungus *Verticillium lecanii*. Ann. Appl. Biol..

[171] Osborne L.S., Landa Z. (1992). Biological control of whiteflies with entomopathogenic fungi. Florida Entomol..

[172] Rashki M., Kharazi-pakdel A., Allahyari H., van Alphen J.J.M. (2009). Interactions among the entomopathogenic fungus, *Beauveria bassiana* (Ascomycota: Hypocreales), the parasitoid, *Aphidius matricariae* (Hymenoptera: Braconidae), and its host, *Myzus persicae* (Homoptera: Aphididae). Biol. Control.

[173] Holler C., Micha S., Schulz S., Francke W., Pickett J.A. (1994). Enemy-induced dispersal in a parasitic wasp. Experientia.

[174] Petersen G., Matthiesen C., Francke W., Wyss U. (2000). Hyperparasitoid volatiles as possible foraging behaviour determinants in the aphid parasitoid *Aphidius uzbekistanicus* (Hymenoptera: Aphididae). Eur. J. Entomol..

[175] Buitenhuis R., McNeil J.N., Boivin G., Brodeuri J. (2004). The role of honeydew in host searching of aphid hyperparasitoids. J. Chem. Ecol..

[176] Gariepy T.D., Messing R. (2012). Development and use of molecular diagnostic tools to determine trophic links and interspecific interactions in aphid-parasitoid communities in Hawaii. Biol. Control.

[177] Kim Y., Kim J. (2003). Biological control of aphids on cucumer in plastic greenhouses using banker plants. Korean J. Appl. Entomol..

[178] Brodeur J., McNeil N. (1994). Life history of the aphid hyperparasitoid *Asaphes volgaris* Walker (Pteromalidae): Possible consequences on the efficacy of the primary parasitoid *Aphidius nigripes* Ashmead (Aphidiidae). Can. Entomol..

[179] Bennison J. (1992). Biological control of aphids on cucumbers: Use of open rearing systems or “banker plants” to aid establishment of *Aphidius matricariae* and *Aphidoletes aphidimyza*. Meded. van Fac. Landbouwwet. Universteit Gent..

[180] McClure T.J., Frank S.D. (2014).

[181] Short M. (2015). Personal Communication.

[182] Desneux N., Denoyelle R., Kaiser L. (2006). A multi-step bioassay to assess the effect of the deltamethrin on the parasitic wasp *Aphidius ervi*. Chemosphere.

[183] Boller E.F., Vogt H., Ternes P., Malavolta C. Working Document on Selectivity of Pesticides (2005). https://www.iobc-wprs.org/ip_ipm/03021_IOBC_WorkingDocumentPesticides_Explanations.pdf.

[184] Cloyd R.A., Soundararajan R.P. (2012). Indirect Effects of Pesticides on Natural Enemies. Pesticides—Advances in Chemical and Botanical Pesticides.

[185] Longley M., Jepson P.C. (1996). Effects of honeydew and insecticide residues on the distribution of foraging aphid parasitoids under glasshouse and field conditions. Entomol. Exp. Appl..

[186] Desneux N., Pham-Delègue M.-H., Kaiser L. (2004). Effects of sub-lethal and lethal doses of lambda-cyhalothrin on oviposition experience and host-searching behaviour of a parasitic wasp, *Aphidius ervi*. Pest Manag. Sci..

[187] Desneux N., Rafalimanana H., Kaiser L. (2004). Dose-response relationship in lethal and behavioural effects of different insecticides on the parasitic wasp *Aphidius ervi*. Chemosphere.

[188] Araya J.E., Araya M., Guerrero M.A. (2010). Effects of some insecticides applied in sublethal concentrations on the survival and longevity of *Aphidius ervi* (Haliday) (Hymenoptera: Aphidiidae) adults. Chil. J. Agric. Res..

[189] Joseph J.-R., Ameline A., Couty A. (2010). Effects on the aphid parasitoid *Aphidius ervi* of an insecticide (Plenum®, pymetrozine) specific to plant-sucking insects. Phytoparasitica.

[190] Zemek R., Jarosik V., Havelka J., Chihale F.P. Effect of host plant and temperature on *Aphidius Colemani* (Hymenoptera: Braconidae) intrinsic rate of population increase. http://www.researchgate.net/publication/267386398_EFFECTS_OF_HOST_PLANT_AND_TEMPERATURE_ON_Aphidius_colemani_%28HYMENOPTERA_BRACONIDAE%29_INTRINSIC_RATE_OF_POPULATION_INCREASE.

[191] Henry L., May N., Acheampong S., Gillespie D.R., Roitberg B.D. (2010). Host-adapted parasitoids in biological control: Does source matter?. Ecol. Appl..

[192] Roux O., Le Lann C., van Alphen J.J.M., van Baaren J. (2010). How does heat shock affect the life history traits of adults and progeny of the aphid parasitoid *Aphidius avenae* (Hymenoptera: Aphidiidae)?. Bull. Entomol. Res..

[193] Zamani A., Talebi A., Fathipour Y., Baniameri V. (2006). Temperature-dependent functional response of two aphid parasitoids, *Aphidius colemani* and *Aphidius matricariae* (Hymenoptera: Aphidiidae), on the cotton aphid. J. Pest Sci..

[194] Kim J.-H., Byeon Y.W., Kim H.-Y., Park C.-G., Choi M.-Y., Han M.-J. (2010). Biological control of insect pests with arthropod natural enemies on greenhouse sweet pepper in winter cropping system. Korean J. Appl. Entomol..

[195] Birch L.C. (1948). The Intrinsic Rate of Natural Increase of an Insect Population. J. Anim. Ecol..

[196] Torres A., Bueno V., Sampaio M.V., de Conti B.F. (2007). Fertility life table of *Aphidius colemani* Viereck (Hymenoptera: Brandonidae, Aphidiinae) on *Aphis gossypii* Glover (Hemiptera: Aphididae). Neotrop. Entomol..

[197] Denis D., Pierre J.S., van Baaren J., van Alphen J.J.M. (2011). How temperature and habitat quality affect parasitoid lifetime reproductive success-A simulation study. Ecol. Modell..

[198] De Koning A.N.M. (1990). Long-term temperature integration of tomato. Growth and development under alternating temperature regimes. Sci. Hortic..

[199] Van den Berg G.A., Butwalda F., Rijpsma E.C. (2001). Practical Demonstration of Mult-Day Temperature Integration.

[200] Gilkeson A. (1987). A note on fecundity of the aphid predator, *Aphidolete aphidimyza* (Rondani) (Diptera: Cecidomyiidae). Can. Entomol..

[201] Mori H., Chant D.A. (1966). The influence of prey density, relative humidity and starvation on the predacious behavior of Phytoseiulus persimilis Athias-Henriot (Acarina: Phytoseiidae). Can. J. Zool..

[202] Van Houten Y.M., van Rijn P.C.J., Tanigoshi L.K., van Stratum P., Bruin J. (1995). Preselection of predatory mites to improve year-round biological control of western flower thrips in greenhouse crops. Entomol. Exp. Appl..

[203] Shipp J.L., Ward K.I., Gillespie T.J. (1996). Influence of temperature and vapor pressure deficit on the rate of predation by the predatory mite, *Amblyseius cucumeris*, on Frankliniella occidentalis. Entomol. Exp. Appl..

[204] Zhang Y., Shipp J.L. (1998). Effect of temperature and vapour pressure deficit on the flight acitivity of *Orius insidiosus* (Hemiptera: Anthrocoridae). Environ. Entomol..

[205] Tuda M., Shima K. (2002). Relative importance of weather and density dependence on the dispersal and on-plant activity of the predator *Orius minutus*. Popul. Ecol..

[206] Yan Y., Chen W. (2012). Effect of humidity on eclosion rate and life of adult *Aphidius gifuensis*.

[207] Fink U., Volkl W. (1995). The effect of abiotic factors on foraging and oviposition success of the aphid parasitoid, *Aphidius rosae*. Oecologia.

[208] Vänninen I., Pinto D.M., Nissinen A.I., Shipp J.L., Johansen N.S. (2012). Prospecting the use of artificial lighting for integrated pest management. Acta Hortic..

[209] Johansen N.S., Vänninen I., Pinto D.M., Nissinen A.I., Shipp L. (2011). In the light of new greenhouse technologies: 2. Direct effects of artificial lighting on arthropods and integrated pest management in greenhouse crops. Ann. Appl. Biol..

[210] Coombe P. (1981). Wavelength specific behaviour of the greenhouse whitefly, *Trialeurodes vaporariorum* (Homoptera: Aleyrodidae). J. Comp. Physiol. A.

[211] Antignus Y., Mor N., Ben J.R., Lapidot M., Cohen S. (1996). Ultraviolet-absorbing plastic sheets protect crops from insect pests and from virus diseases vectored by insects. Environ. Entomol..

[212] Doukas D., Payne C.C. (2007). The use of ultraviolet-blocking films in insect pest management in the UK; effects on naturally occurring arthropod pest and natural enemy populations in a protected cucumber crop. Ann. Appl. Biol..

[213] Shipp J.L., Johansen N., Vänninen I., Jacobson R. (2011). Greenhouse climate: An important consideration when developing pest management programs for greenhouse crops. Acta Hortic..

[214] Vanninen I., Pinto D., Nissinen A., Johnansen N.S., Shipp L. (2010). Prospecting the use of artifical lighting for integrated pest management. ISHS Acta Horticulturae.

[215] McClure M., McNeil J.N. (2009). The effect of abiotic factors on the male mate searching behavior and the mating success of *Aphidius ervi* (Hymenoptera: Aphidiidae). J. Insect Behav..

[216] Marchand D., McNeil J.N. (2000). Effects of wind speed and atmospheric pressure on mate searching behavior in the aphid parasitoid *Aphidius nigripes* (Hymenoptera: Aphidiidae). J. Insect Behav..

[217] Ventilation for Greenhouses. https://ag.umass.edu/fact-sheets/ventilation-for-greenhouses.

